# Native Collagen and Total Lipid Extract Obtained from *Caranx hyppos* By-Products: Characterization for Potential Use in the Biomedical and Nutraceutical Fields

**DOI:** 10.3390/md23110432

**Published:** 2025-11-09

**Authors:** Sheyza Menéndez-Tasé, Evelin Gaeta-Leal, Darío Iker Téllez-Medina, Daniel Tapia-Maruri, Edgar Oliver López-Villegas, Georgina Calderón-Domínguez, Tzayhri Gallardo-Velázquez, Guillermo Osorio-Revilla, Mayuric Teresa Hernández-Botello, Diana Maylet Hernández-Martínez

**Affiliations:** 1 Departamento de Biofísica, Escuela Nacional de Ciencias Biológicas (ENCB), Instituto Politécnico Nacional, Prolongación de Carpio y Plan de Ayala s/n, Col. Santo Tomás, Alcaldía Miguel Hidalgo, Mexico City 11340, Mexico; sheyzamenendeztase@gmail.com (S.M.-T.); eveibq@gmail.com (E.G.-L.); gtzayhri@yahoo.com (T.G.-V.); mthernandezbo@ipn.mx (M.T.H.-B.); 2 Departamento de Ingeniería Bioquímica, Escuela Nacional de Ciencias Biológicas (ENCB), Instituto Politécnico Nacional, Av. Wilfrido Massieu Esq. Cda. Miguel Stampa s/n, Alcaldía Gustavo A. Madero, Mexico City 07738, Mexico; dtellez@ipn.mx (D.I.T.-M.); gcalderon@ipn.mx (G.C.-D.); osorgi@gmail.com (G.O.-R.); 3 Laboratorio de Microscopía Avanzada, Centro de Desarrollo de Productos Bióticos (CEPROBI), Instituto Politécnico Nacional, Carretera Yautepec-Jojutla km. 8.5, San Isidro Yautepec, Morelos 62731, Mexico; dmaruri@ipn.mx; 4 Laboratorio Central de Microscopía, Departamento de Investigación-SEPI, Escuela Nacional de Ciencias Biológicas (ENCB), Instituto Politécnico Nacional, Prolongación de Carpio y Plan de Ayala s/n, Col. Santo Tomás, Alcaldía Miguel Hidalgo, Mexico City 11340, Mexico; ivoliver@hotmail.com

**Keywords:** crevalle jack, fish residues, collagen acid extraction, physicochemical analysis, structural analysis, omega-3 fatty acids, lipid antioxidant capacity

## Abstract

The processing of fishery products generates a substantial amount of by-products, which can be utilized to promote a circular economy. The objective of the present study was to extract and characterize native collagen and total lipid extract from the fish skin and bones of crevalle jack (*Caranx hippos*). Physicochemical, structural, and morphological properties were evaluated for collagens. Chemical composition and functional properties were evaluated for lipid extracts. Native type I collagens were obtained by acid extraction, yielding approximately 2.64–6.16% (d.b.). The elemental chemical analysis showed its purity. The stability of the triple helix of collagen was verified through characteristic bands in the FTIR and UV spectra, the peaks at 2θ, around 7.5° and 19.5° obtained by XRD, and the bands of SDS-PAGE. Collagens show isoelectric points of 4.94 (skin) and 4.90 (bone), thermal stabilities of 53.40 °C (skin) and 46.88 °C (bone), and the percentage surface porosities of 41.28 (skin) and 38.84 (bone), all of which demonstrate their potential as a raw material in the biomedical field. The total lipids obtained were extracted using the Soxhlet and Folch methods. The extracts show EPA (1.26–3.16%) and DHA (3.94–9.78%) contents, with inhibition percentages of 32.7% (ABTS), 19.6% (DPPH), and 70.83% (β-carotene). These results highlight the potential of total lipid extract for nutraceutical and food applications.

## 1. Introduction

According to the FAO, there is rapid annual growth in per capita consumption of aquatic foods [1]. Fish packing companies and distribution centers generate considerable quantities of waste, including heads, viscera, skeletons, skin, and scales. The by-products can represent between 30 and 70% of the fish on a wet basis [2]. The recovery of fishery by-products is a viable alternative for reducing waste, preventing it from becoming a source of pollution, and generating raw materials with applications in various industries. This action is important, as the sustainable use of oceans, seas, and marine resources is a key goal of the UN’s 2030 Agenda for Sustainable Development [3]. 

Among the bioactive compounds extracted from marine by-products are collagen and fish lipids. 

Collagen is of interest to multiple industries, including food, biomedical, pharmaceutical, and cosmetics [4,5]. Collagen is a structural and fibrous protein with high biomechanical resistance and protective capacity, forming part of connective tissue [6]. It is an abundant protein in skin and bone, and due to its solubility, it is classified as a water-insoluble protein [7]. Native collagen has an intact triple helix structure in which three polypeptide chains rich in glycine (Gly), proline (Pro), and hydroxyproline (Hyp) are assembled [5]. There are 29 types of collagens, but type I is used in the biomaterials industry [5]. There are two main chains of type I collagen, designated as alpha 1 (α1) and alpha 2 (α2), with minor differences in their amino acid composition [8]. 

Traditionally, collagen has been extracted from cattle and pigs, as well as from poultry by-products [9]. Fish collagen has some advantages, such as greater biocompatibility and biodegradability [10]. Additionally, there are no reports of transmission of potentially transmissible zoonotic diseases such as transmissible spongiform encephalopathy (TSE) or foot-and-mouth disease (FMD) [11]. Fish collagen is also less likely to trigger allergic reactions [9]. Moreover, there are no cultural or religious restrictions for Muslim, Jewish, or Hindu communities [11,12]. Due to these concerns, obtaining collagen from marine organisms has been presented as an alternative. According to published reports, the global marine collagen market is expected to have a Compound Annual Growth Rate (CAGR) of approximately 7.4% between 2024 and 2030 [13].

On the other hand, obtaining lipids from marine sources is important due to their high content of omega-3 polyunsaturated fatty acids (PUFAs), such as EPA and DHA. PUFAs are commonly obtained from fatty fish such as tuna, salmon, sardines, and anchovies, as well as from shellfish and algae [14].

The extraction of lipids from fish processing by-products optimizes the resource utilization. It promotes sustainability and the circular economy, as the nutritional value of fish waste is almost identical to that of edible parts in terms of vitamins, proteins, and lipids [15]. Total lipid fatty acids from fish are a high-value-added raw material due to their benefits for cardiovascular health, the immune system, the treatment of neuropsychiatric disorders, and the growth and development of infants [16,17]. This is why there is growing interest in enriching food supplements with omega-3 fatty acids [16]. According to published reports, the global marine omega-3 market is projected to grow at a compound annual growth rate (CAGR) of approximately 9.1% between 2025 and 2030 [18].

A species of fish from the Carangidae family with high potential for exploitation is the crevalle jack (*Caranx hippos*). The crevalle jack is a carnivorous, pelagic, and migratory species with a wide geographical distribution in the Atlantic Ocean. It is found from Nova Scotia to Uruguay and from Portugal to Angola [19]. The species is characterized by its growing demand in the global market [20] and its availability at low retail prices ranging from $2.20 to $5.30 per kilogram [21]. Adult crevalle jacks are morphologically distinguished by a large head, which accounts for nearly a third of their body, and smooth skin with small scales. Fisheries waste is often undervalued, as it is sent directly to composting, incineration, or landfilling, and only a small portion is processed to obtain fish meal or fish oil [4,22]. However, it can serve as a source of valuable raw materials. 

There is information on collagen extraction processes [23,24] and total lipid fatty acids [25,26] from by-products of various fish species. However, there are currently no articles on the use of crevalle jack (*Caranx hippos*) waste, despite being a species widely distributed in the Atlantic Ocean, and it is very inexpensive. Therefore, the valorization and characterization of *Caranx hippos* by-products represent an opportunity to obtain materials with the required characteristics in the pharmaceutical, biomedical, and food industries.

This study aimed to utilize the skin and bones of crevalle jack (*Caranx hippos*) to obtain native collagen and omega-3-rich lipids. Native collagen was evaluated for its physicochemical, structural, and morphological properties. On the other hand, total lipid extracts were characterized using infrared spectroscopy, fatty acid profile analysis, and antioxidant capacity assays. This study aims to explore crevalle jack by-products as raw materials with remarkable functional properties for possible applications. In the biotechnology field, collagen is beneficial for forming scaffolds. Regarding fish oil, it is helpful in the nutraceutical field.

## 2. Results

The skin and bone waste from crevalle jack (*Caranx hippos*) was characterized, and collagen and total lipid extract were subsequently obtained. These biomaterials were then characterized. The results are as follows.

### 2.1. Chemical Composition of Caranx hippos By-Products

In the *Caranx hippos*, the edible percentage (fillets) was 37.22 ± 5.12%. The remaining 62.78 ± 13.64% constitutes waste. In the present study, the waste of interest represented 5.35 ± 1.41% (skin) and 36.48 ± 9.14% (bone) of the fish.

The skin chemical composition was 48.84 ± 0.42% moisture, 31.10 ± 0.13% protein, 17.46 ± 2.24% lipids, 2.56 ± 0.02% ash, and 0.04 ± 0.00% carbohydrates. Moreover, the bone chemical composition was 54.77 ± 0.68% moisture, 10.61 ± 0.06% protein, 14.19 ± 0.73% lipids, 21.92 ± 0.38% ash, and 0.00 ± 0.00% carbohydrates. 

The hydroxyproline (Hyp) content from skin and bone on a wet basis was 6.16 ± 0.37 mg/g and 4.38 ± 0.74 mg/g, respectively; on a dry basis, it was 12.03 ± 0.01 mg/g and 9.68 ± 0.13 mg/g, respectively. 

### 2.2. Collagen Extraction Yield

Native freeze-dried collagen was obtained from the skin (SC) and bone (BC) of *Caranx hippos* through acid extraction. The experimental yields of SC and BC on a wet basis were 3.15 ± 0.12% and 1.19 ± 0.2%, respectively; on a dry basis, they were 6.16 ± 0.18% and 2.64 ± 0.48%, respectively.

### 2.3. Chemical Characterization of Collagen

Table 1 shows the moisture, ash, and Hyp content values for SC and BC. Both collagens showed significant differences (*p* < 0.05) in the parameters evaluated.

### 2.4. Element Composition Analysis of Collagen

**Figure 1:**
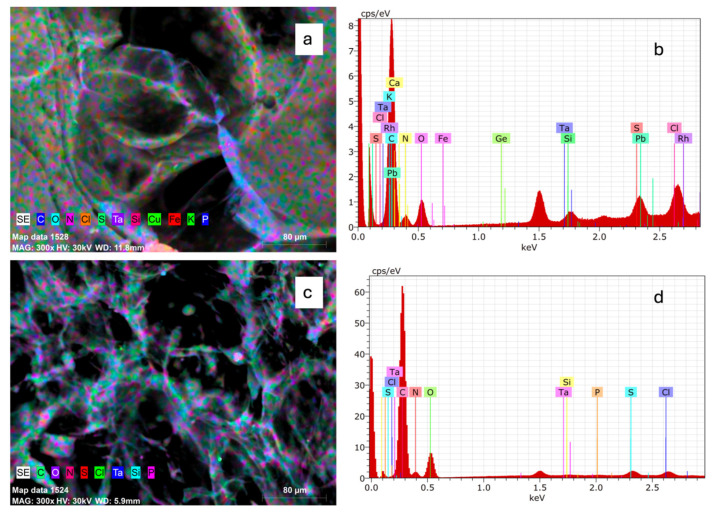
SEM-EDX mapping images and EDS spectra of collagen from *Caranx hippos*. SC, skin collagen (**a**,**b**); BC, bone collagen (**c**,**d**).

Elemental chemical analysis by Scanning Electron Microscopy-Energy-Dispersive X-ray Spectroscopy (SEM-EDX) of the surface of SC and BC showed the minerals listed in Table 2. Figure 1 shows SEM-EDX results. Figure 1a,c represent the combination of SEM micrographs and EDX maps of SC and BC, respectively. Figure 1b,c represent EDX spectra of SC and BC, respectively.

### 2.5. Physicochemical Characterization of Collagen

#### 2.5.1. Color of Collagen

Table 1 shows the CIELAB color parameters (L*, a*, b*), color index (CI), whiteness (W), and Chroma (C*) for SC and BC. The collagens showed significant differences (*p* < 0.05) for color parameters.

#### 2.5.2. Zeta Potential (ζ) of Collagen

**Figure 2:**
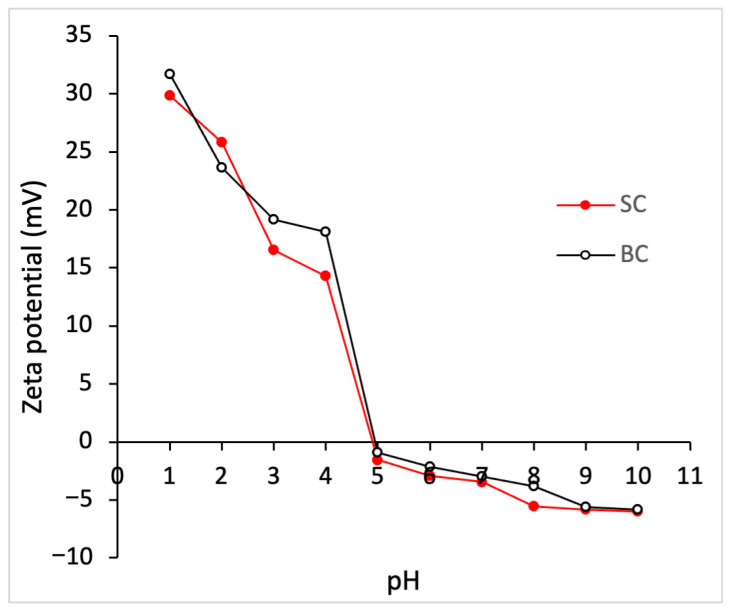
Zeta potential of collagen from *Caranx hippos*. SC, skin collagen; BC, bone collagen.

Figure 2 shows the zeta potential of SC and BC at different pH values. The isoelectric point (pI) for SC and BC was 4.94 and 4.90, respectively. It was observed that at pH values lower than the pI, the zeta potential acquires a positive value, while at higher pH values, relatively stable and negative values are observed. There is no statistically significant difference (*p* ≥ 0.05) between the zeta potential values of SC and BC. 

#### 2.5.3. Differential Scanning Calorimetry (DSC) of Collagen

**Figure 3:**
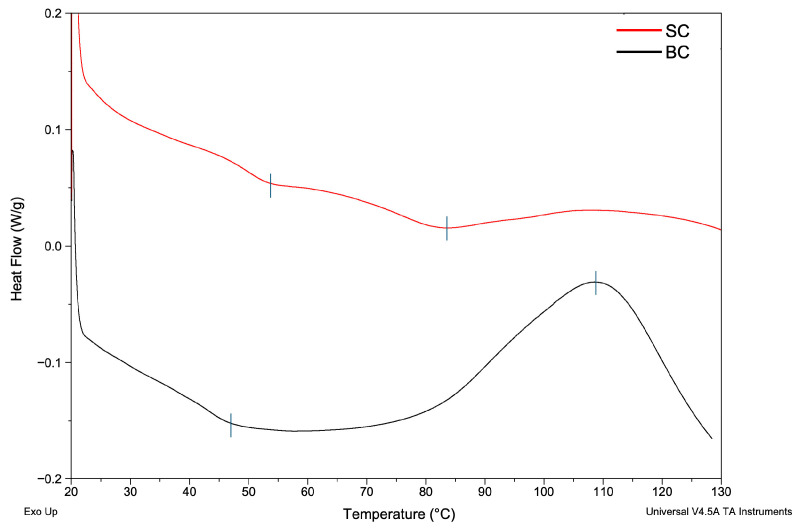
Differential scanning calorimetry diagram of collagen films from *Caranx hippos*. SC, skin collagen; BC, bone collagen. Exo Up means exothermic peaks are shown as upward peaks in the thermograms. Blue vertical line indicates the temperature of thermal transitions.

In this study, thermal stability was evaluated using DSC. Table 3 shows the temperatures and enthalpies obtained for films made with SC and BC. Figure 3 shows the DSC diagram.

The results showed that the thermal transition differs between the two samples. This indicates that the type of tissue influences the thermal behavior of the collagen film during analysis (*p* < 0.05).

### 2.6. Structural Characterization of Collagen

#### 2.6.1. Fourier Transform Infrared (FTIR) Spectra of Collagen

**Figure 4:**
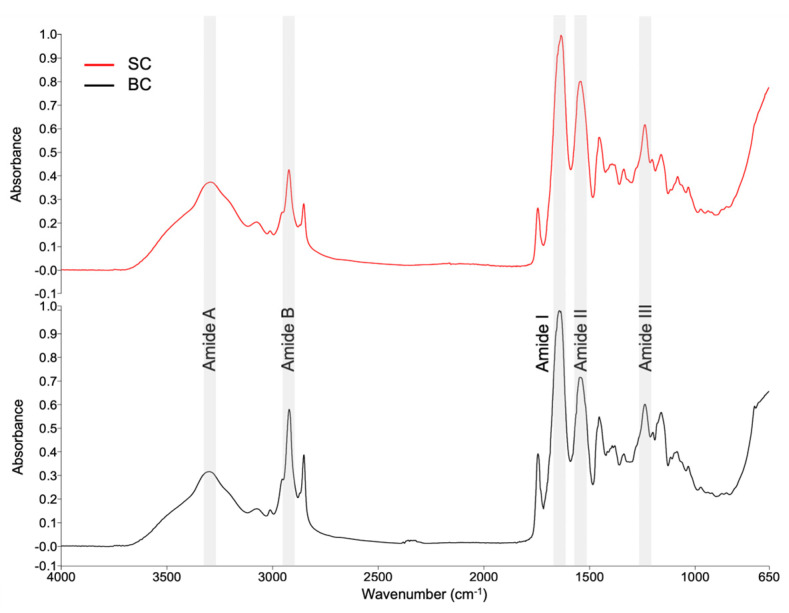
FTIR spectra of collagens from *Caranx hippos*. SC, skin collagen; BC, bone collagen.

Figure 4 shows the SC and BC spectra in the range of 4000–650 cm^−1^ obtained by FTIR spectroscopy. 

Comparing the spectra of the two types of collagens yielded a correlation coefficient of 0.945. It demonstrates the degree of similarity between the two spectra. The correlation was obtained using the Compare tool in the Spectrum software (version 10.5.3.738, PerkinElmer, Inc., USA). A value of 1 indicates that the spectra are identical.

Table 4 shows a summary of the bands and respective assignments. Collagen is usually identified by the presence of five amide bands: amide A, amide B, amide I, amide II, and amide III [27,28]. According to the analysis of variance, there are no statistically significant differences (*p* ≥ 0.05) between the positions of the bands in SC and BC.

#### 2.6.2. UV Spectra of Collagen

**Figure 5:**
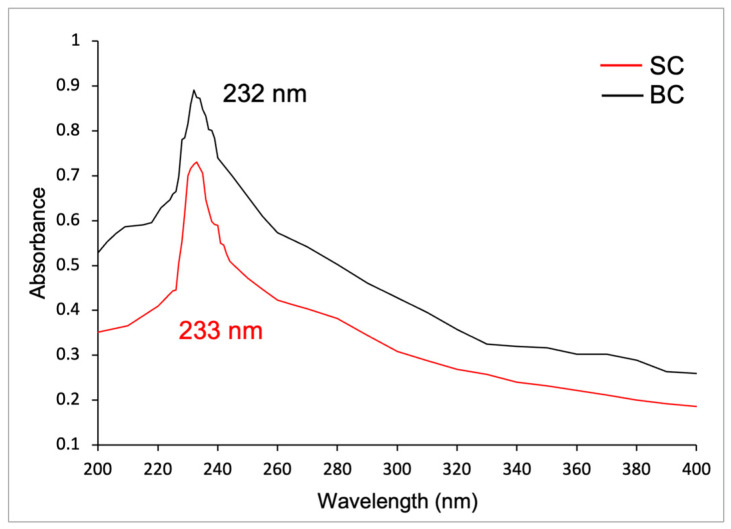
UV spectra of collagens from *Caranx hippos*. SC, skin collagen; BC, bone collagen.

Figure 5 shows the UV spectra of SC and BC. The SC presented a maximum absorbance of 0.731 at a wavelength of 233 nm in the UV region. The BC presented a maximum absorbance of 0.891 at 232 nm. According to the analysis of variance, there are no significant differences between them (*p* ≥ 0.05). The first-derivative UV spectra are presented in Figure S1 of the supplementary material.

#### 2.6.3. X-Ray Diffraction (XRD) Analysis of Collagen

**Figure 6:**
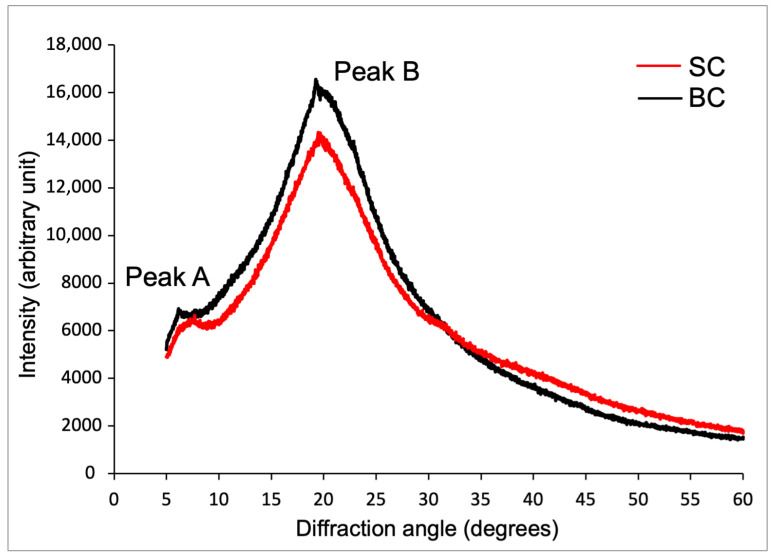
X-ray diffraction diagram of collagens from Caranx hippos. SC, skin collagen; BC, bone collagen.

Figure 6 shows the X-ray diffractograms of SC and BC from *Caranx hippos*. Both collagens exhibited two characteristic peaks (labeled A and B) located at 7.70° and 19.96° for SC and 7.30° and 19.22° for BC, respectively. 

For SC and BC, the interlaminar distance (dÅ) result for peak A was 11.46 Å and 12.09 Å, respectively; and for peak B, it was 4.42 Å and 4.59 Å, respectively.

#### 2.6.4. Sodium Dodecyl Sulfate Polyacrylamide Gel Electrophoresis (SDS-PAGE) of Collagen

**Figure 7:**
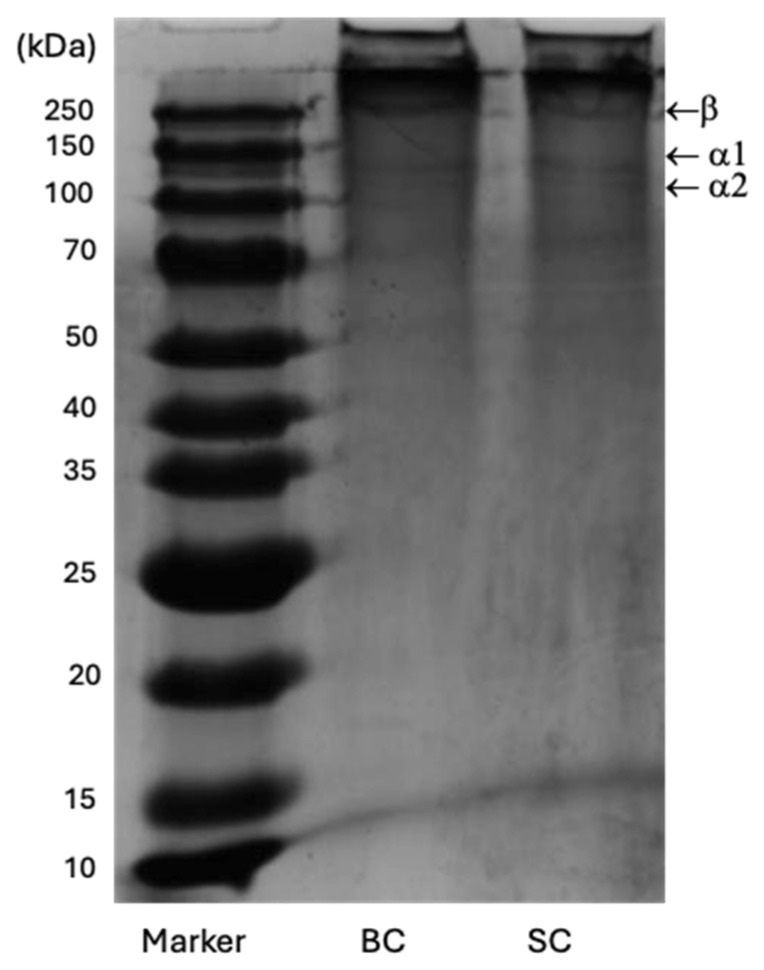
SDS-PAGE profiles of SC and BC from *Caranx hippos*. Line 1: Standard protein marker; Line 2: BC; Line 3: SC.

Figure 7 shows the SC and BC electropherogram. The gel revealed three bands, each with a different electrophoretic mobility. A first band of high intensity was identified, followed by two faint bands, suggesting differences in their molecular weights, although all were greater than 100 kDa (Table 5).

The collagen SC and BC have a similar electrophoretic profile, consisting of two α chains (α1 and α2) and a β dimer that has a higher molecular weight than the α chains. The union of two α1 chains forms the β chain through intramolecular crosslinking [12]. 

### 2.7. Environmental Scanning Electron Microscopy (ESEM) of Collagen

**Figure 8:**
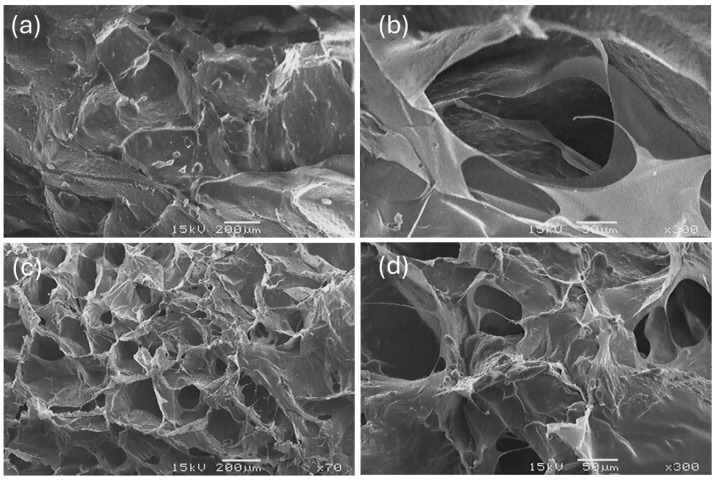
ESEM images of SC (**a**,**b**) and BC (**c**,**d**) of *Caranx hippos* (×70, ×300).

Figure 8 shows ESEM micrographs of SC and BC. The collagens have a rough and irregular surface, with pores of different sizes and a multidirectional formation. This morphology has been identified in the literature consulted for freeze-dried collagens from other marine raw materials [27,31,33,34].

Table 6 shows the results of surface porosity percentage and pore diameter for SC and BC calculated using ImageJ software.

### 2.8. Total Lipid Extraction Yield

The total lipids from the skin (SLs) and bone (BLs) of the crevalle jack (*Caranx hippos*) were extracted using the Soxhlet method. The wet basis (w.b.) yield for SLs and BLs was 17.46 ± 2.24% and 14.19 ± 0.73%, respectively, and the dry basis (d.b.) yield was 29.26 ± 7.24% and 31.37 ± 1.83%, respectively. It should be noted that SL was also extracted using the Folch method (SLf), which is a cold method, yielding 4.22 ± 2.03% (w.b.) and 7.07 ± 3.4% (d.b.).

### 2.9. Fourier Transform Infrared (FTIR) Spectra of Total Lipid Extracts

**Figure 9:**
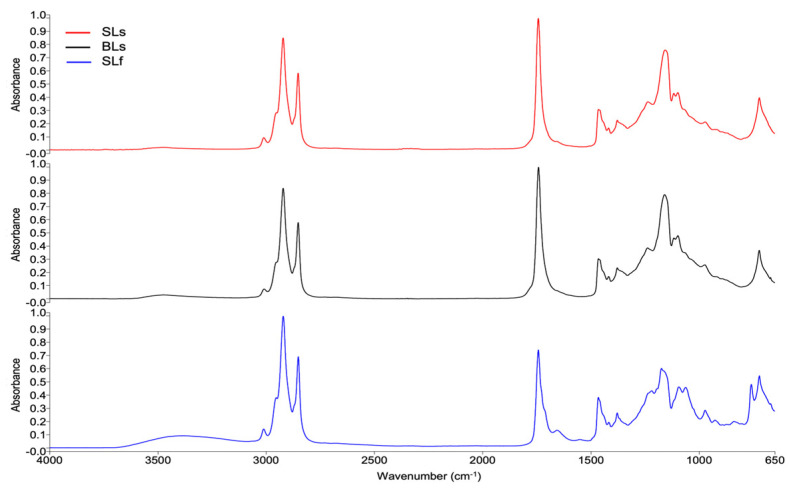
FTIR spectra of total lipid extracts from *Caranx hippos*. SLs, skin lipids (Soxhlet); BLs, bone lipids (Soxhlet); SLf, skin lipids (Folch).

Figure 9 shows the FTIR spectra of SLs and BLs extracted by the Soxhlet method and the SLf sample extracted by the Folch method. Table 7 shows a summary of the bands and their respective assignments. The Compare tool of the Spectrum software was used to compare the spectra. A correlation of 0.974 was obtained between SLs and SLf, indicating a high overall similarity between the samples, but also suggesting differences in composition depending on the extraction method.

### 2.10. Total Lipid Fatty Acids

Table 8 shows the total lipid fatty acid (fatty acids derived from lipid extract) found in SLs, BLs, and SLf, as well as in a sample of commercial fish oil (CFO). The relationships between fatty acid groups obtained for the *Caranx hippos* samples are also shown.

### 2.11. Antioxidant Capacity of Total Lipid Extract

Table 9 shows the antioxidant capacity of SLf and a sample of commercial fish oil (CFO). It should be noted that only SLf was evaluated, as it had the best fatty acid profile.

The results are expressed using three tests. The β-carotene bleaching test, a specific method for evaluating the antioxidant activity of lipophilic compounds, simulates oxidation conditions more closely resembling those that occur in real matrices [35,36]. The ABTS assay was also employed, which enables the measurement of the activity of both hydrophilic and lipophilic compounds in complex samples, such as oils [36]. The DPPH assay is mainly oriented towards lipophilic compounds that can only be dissolved in organic solvents [36].

## 3. Discussion

### 3.1. Chemical Composition of Caranx hippos By-Products

The skin and bones of *Caranx hippos* represent a high percentage (41.83 ± 10.55%) of usable waste. Although the percentage of skin is low (Table 1), the hydroxyproline (Hyp) content was higher in the skin than in the bone. The determination of Hyp has been used as a method to quantify the amount of collagen in a particular tissue, since this amino acid is almost exclusive to collagens, being present in insignificant amounts in other proteins [37]. For this reason, both skin and bones could be used for the extraction of marine collagen in a comparable manner.

### 3.2. Yield and Chemical Characterization of Collagen

The SC yield was similar to that reported for collagen extracted in acetic acid (ASC) from *Priacanthus tayenus* skin [12] and *Reinhardtius hippoglossoides* skin [28], with values of 5.79% (d.b.) and 3.8% (w.b.), respectively. However, the yield was higher than that obtained for *Gadus morhua* skin with a value of 1.5% (w.b.) [38]. Meanwhile, the BC yield is comparable to that reported for acid extraction from the skulls and spines of *Katsuwonus pelamis*, with values of 2.47 and 3.57% (d.b.), respectively [39]. Other authors reported that ASC yield values ranged from 2.6 to 61.26% and 0.28 to 13.68% (d.b.) for skin and bone, respectively, for different species [40]. The yield variation is attributed to differences in extraction conditions and structural differences in the collagen of fish [41]. 

The collagen is bioactive and biodegradable. It also possesses mechanical strength, flexibility, low immunogenicity, and weak antigenicity. Due to these characteristics, native collagen is used in biomedical applications such as regenerative medicine, tissue engineering, and wound healing [42,43]. For instance, the collagen processing allows the creation of scaffolds of various sizes and shapes, such as hydrogels, sponges, 3D composites, and fibrous mats [38,43]. To utilize the collagen and meet the requirements of the final product, it is necessary to verify the chemical composition and purity of the collagen, as well as to understand its molecular structure, stability, and morphology [7]. 

The moisture content in collagen should be determined, as it affects the stability and processability of collagen. In both types of collagen, the value was less than 1.3%. The ash content of SC was significantly lower (*p* < 0.05) than that of BC, which can be attributed to the mineralization of the bone matrix. Even so, the ash content is very low, indicating that impurities and inorganic matter were removed during extraction.

The Hyp content of SC (19.02 mg/g collagen) was higher than that of BC (15.47 mg/g). A higher proportion of collagen in the skin compared to bone has been previously reported [41,44]. The SC Hyp values were lower than those reported for collagen extracted in acetic acid (ASC) from the skin of *Priacanthus tayenus* (64.5 ± 0.92 mg HPro/g collagen) [12] and *Sphyraena* sp. (82.78 ± 0.19 mg HPro/g collagen) [33]. The BC Hyp result was lower than those reported from the bone of *Priacanthus tayenus* (42.5 ± 0.94 mg HPro/g collagen) [45] and *Saurida tumbil* (84.85 ± 1.38 mg HPro/g collagen [44]. The variation in hydroxyproline content between species may depend on the species, environment, and body temperature of the fish [45]. It should be noted that higher Hyp content is related to a higher denaturation temperature, as Hyp forms hydrogen bonds between collagen polypeptides, thereby stabilizing the triple helix [37].

Based on elemental chemical analysis, it was found that for SC and BC, the sum of carbon, oxygen, and nitrogen represented 96.42% and 98.66%, respectively, which is understandable given the protein composition. All other elements are found in low concentrations. The presence of Na and Cl may be due to the use of NaCl in collagen precipitation during the extraction process [46]. The presence of the element S may be due to the presence of cysteine, an amino acid that contains sulfur [46]. The iron present in SC may be because the gill membrane and intestinal mucosa absorb soluble iron from water, and some capillary vessel residues remain attached to the skin during the cleaning process [47]. Lead (Pb), which is a heavy metal, was found only in SC. *Caranx hippos* is a carnivorous species, so the presence of lead could be explained by bioaccumulation. An interesting finding is the presence of tantalum (Ta) and rhodium (Rh), as they are not common elements. The element Ta is reported to have excellent chemical stability and compatibility with living organisms [48]. It should be noted that SEM-EDX analysis is semi-quantitative for trace elements.

### 3.3. Physicochemical Characterization of Collagen

Concerning physicochemical properties, color is a crucial attribute in collagen, as it enables potential applications in the pharmaceutical and cosmetic industries, where a lighter color may be preferable to avoid imparting color to finished products. In the study, SC had a less bright color than BC. Therefore, the color parameters between the two types of collagens showed significant differences (*p* < 0.05), which could be due to the pigments present in the skin of *Caranx hippos*. An alternative for removing color could be to use a hydrogen peroxide solution as a bleaching agent [40].

Collagen has a triple helix structure rich in ionizable groups (amino, carboxyl, imidazole, and hydroxyl), which makes pH affect the state of charge of different peptide chains. The zeta potential, also called the electrokinetic potential, reflects the net surface charge with which a collagen molecule moves in a medium. Therefore, zeta potential values allow the pH at which the protein has a net zero charge to be identified, which corresponds to the isoelectric point (pI) [31]. According to Yue et al. (2024) [49], collagen at pI tends to curl, showing the minimum swelling and the minimum viscosity due to the proximity of the peptide chains. When the pH is below the pI, the molecule is protonated, and above the pI, it is deprotonated. A high surface charge, whether positive or negative, generates intermolecular repulsions that tend to stretch the collagen molecular chain, due to side groups on the collagen peptide chain repelling each other due to the same charge [49]. Thus, the change in pH affects the collagen’s structure, solubility, denaturation temperature, and crystalline structure, determining its potential uses. For example, by varying the pH of the solution away from the pI, the distance between the peptide chains increases, causing varying degrees of swelling. Moreover, a pH close to the physiological value (7.4) favors the self-assembly of collagen in the formation of fibrils [49].

Collagen extracted from various raw materials exhibits different surface charges and pI values due to its specific amino acid composition [9]. The isoelectric points of SC (4.94) and BC (4.9) were found at acidic pH, possibly due to the higher density of carboxyl groups [50]. In the literature, pI values comparable to those obtained were reported for collagen from the skin of *Pangasianodon hypophthalmus*, with a value of 4.27 [51], and for collagen from the scales of *Parupeneus heptacanthus*, with a value of 4.96 [52]. The pI of other marine by-products ranges between 7.26 and 4.96 [9].

Regarding thermal properties, two thermal transitions were observed in the SC and BC films. The first endothermic peak, 53.40 °C (SC) and 46.88 °C (BC), is related both to the denaturation temperature (Td) of collagen in the film, which involves the breakdown of its triple helical structure [7], and to the loss of bound water [53]. In the SC film, denaturation occurs at a higher temperature than in the BC film, suggesting greater thermal stability. This finding was in accordance with the high Hyp content of SC. In BC, the lower Hyp content indicates a lower number of hydroxyl groups participating in the formation of hydrogen bonds. Hydrogen bonds help hold the triple helix structures together within the collagen molecule [44]. That is why the Hyp content decrease represents a loss of thermal stability in collagen.

The variation in thermal transition or denaturation temperature depends on the raw material, extraction method, chemical composition of collagen, and habitat temperature where the fish live [53]. The first peak (Td) is comparable to results reported for ASC collagen in a dry state. The Td was 51.59 °C for *Oreochromis niloticus* [54]. Thermal events of 65.9, 70.9, and 74.9 °C have been reported for collagen films from *Cynoscion acoupa*, *Arius parkeri*, and *Cynoscion leiarchus*, respectively [55]. Moreover, an endothermic peak of 79.3 °C was reported for collagen films from *Esox lucius* scales [53].

In the thermogram of the SC film, the second thermal event reflects a more advanced structural change, possibly related to the cross-linked part of collagen and destruction of the collagen film [53].

In the thermogram of the BC film, the exothermic peak can be attributed to the recrystallization of denatured collagen, a process that involves the rearrangement of collagen’s cross-linked structure, releasing energy as a common effect in polymers [56].

Bone is a crystalline polymer that combines collagen with hydroxyapatite domains. Hydroxyapatite residues in BC could also be the cause of differences in the thermal behavior of SC and BC. Vladu et al. (2025) [57] reported an endothermic peak followed by an exothermic peak in collagen/hydroxyapatite scaffolds. They associated the exothermic peaks between 135 and 400°C with the loss of structural water from collagen, partial denaturation due to degradation of polypeptide chains, and partial oxidation of the fragments.

These denaturation temperatures suggest that collagens exhibit good thermal stability above 37 °C (the basal human body temperature), which could make them useful in biomedical applications where resistance to specific temperature ranges is required [29].

### 3.4. Structural Characterization of Collagen

The structural evaluation of collagen is crucial for understanding its molecular organization, which in turn influences its function. Various techniques are employed for this purpose, including FTIR and UV-vis spectroscopy, XRD, and SDS-PAGE.

The FTIR spectra obtained for SC and BC exhibit five bands, representing characteristic functional groups in collagen (Table 4), and are comparable to those reported in the literature [12,27,30,32].

In both collagens, the position of the amide A band appears to have shifted to lower wavenumbers than 3400 [31,34]. This decrease suggests that N-H in peptide bonds was involved in stabilizing the collagen triple helix structure through hydrogen bonding [32]. The amide B is related to C-H asymmetric stretching in the methyl group [29]. The amide I, amide II, and amide III bands are directly associated with the secondary structure of collagen. The appearance of these bands in the spectra indicates the existence of a triple helical structure [27,32,34].

To verify whether the triple helix is still present, the relationship between the absorption of amide III and the peak near 1450 cm^−1^ is established (A_III_/A_1450_). If the ratio is close to 1, this indicates that the triple helix structure is preserved [27,44]. In the present study, ratios of 1.09 and 1.11 were obtained for SC and BC, respectively, indicating the integrity of the triple helix structure. The integrity of the triple helix is essential for collagen to have adequate mechanical rigidity and enzymatic stability [7].

On the other hand, in the UV spectrum of SC and BC, according to Jaziri et al. (2022) [44] and Liao et al. (2018) [58], the maximum absorption peak at 230 nm is associated with the functional groups of carbonyl (C=O), carboxyl (-COOH), and amides (CONH_2_) which belong to the polypeptide chains of collagen. In contrast, other amino acids and proteins absorb UV radiation around 250 and 280 nm. The findings of these authors align with the results of this study [28,44,58].

This maximum absorbance in the ultraviolet region also aligns with the results reported by other authors, who found values of 230.30, 230.90, and 231.9–233.9 nm for collagen of *Lates calcarifer* [58], *Oreochromis niloticus* [58], and *Saurida tumbil* [44], respectively. 

The diffraction pattern (XRD) is related to the diameter of the triple helix collagen molecule and the single left-handed helix chain in the collagen structure [46].

Peak A, which is smaller and more rounded, is associated with the diameter of the triple helix structure (1.40 nm), and its intensity is directly related to the content of this structural conformation, which always appears around 7°. Peak B, which is more acute in shape, correlates with the distance between adjacent amino acid residues or molecular chains (0.44 nm) along the collagen helix and is located near 20–22° [32,59]. The dÅ values were in accordance with the reported values from other species [33,44,60]. For *Caranx hippos*, the higher dÅ values obtained for BC suggest lower crystallinity than SC, and explain the lower Td for BC in the DSC analysis. The higher dÅ is explained by a lower amount of intermolecular and intramolecular bonds (such as hydrogen bonds) that stabilize the collagen structure. This implies greater repulsive forces in collagen at a pH value far from the pI.

The results indicate that SC and BC exhibit an ordered structure, are not denatured, and possess a molecular organization compatible with collagen type I, which is consistent with the findings of FTIR spectroscopic characterization. These results also agree with the characteristic diffraction peaks of collagen reported by other authors [44,58,60].

SDS-PAGE analysis provides information on the purity and structure of collagen polypeptide chains. The subunit composition formed by two alpha chains (α1 and α2) at about 100 kDa, and the β dimer at around 250 kDa in the electropherograms of SC and BC indicated a type I collagen pattern, similar to those reported by other authors for different fish species [12,33,61]. Type I collagen is the most common pattern in fish [12] and is commonly used in tissue engineering as a biomaterial for bone scaffolds [4].

There is no presence of subunits or weak α chains smaller than 100 kDa, which corroborates that there was no partial hydrolysis of collagen components, indicating the structural integrity of the collagens obtained. It also indicates the absence of non-collagenous proteins, considering the purity of the extracts represented [28].

The intensity ratio of β bands/(α1 + α2) in BC and SC was 0.64 and 0.61, respectively. The β chain degraded into α1 and α2 chains by 60.82% and 61.90%, respectively, for BC and SC. This modification may be due in part to the extraction conditions with 0.5 M acetic acid. These results coincide with those reported by Tan & Chang (2018) [62] for collagen extracted with acetic acid.

The surface morphology of BC and SC collagen was studied using ESEM. Both collagens exhibit porous areas and compact, plate-shaped conglomerates, the latter being more pronounced in SC. The micrographs obtained show a higher degree of fiber packing compared to images reported by other researchers [31]. This phenomenon occurs due to the presence of pyridinoline in the collagen molecule, since the presence of pyridinoline is the main molecule responsible for collagen crosslinking [63].

The space between the intertwined sheets observed in the micrographs represents collagen porosity, a property that can facilitate the incorporation of bioactive compounds or drugs [64]. This characteristic is useful in biomedical applications, such as the construction of collagen matrix scaffolds for tissue repair engineering. The structural properties of this material, including pore and fiber distribution, wall morphology, and interconnectivity, directly influence cell behavior, such as adhesion, growth, migration, differentiation, gene expression, and tissue formation [34].

BC had a higher number of pores than SC, although with slightly lower surface porosity. This difference can be attributed to a greater presence of small pores in BC, whereas SC collagen tends to form larger, more porous structures, albeit in smaller quantities. Despite these descriptive differences, no statistically significant differences were observed between SC and BC in any of the parameters evaluated (*p* ≥ 0.05). This result could be attributed to both the structural similarity between the two types of collagens and the small sample size.

In the study by López (2019) [65], scaffolds were developed from marine ASC from the skin of *Prionace glauca* and calcium phosphate from the teeth of the same species. Several chemical cross-linkers were used to reinforce the polymer structure. As a result, porosity percentages ranging from 48.40 ± 14.80% to 87.2 ± 1.30% were obtained, depending on the amount of cross-linking agent incorporated into the scaffold formulation.

The results obtained in this study showed that SC and BC achieved considerable surface porosity percentages, even without the addition of cross-linking agents. This finding suggests that marine collagen from *Caranx hippos*, in its native form, may represent a promising raw material for designing porous scaffolds in tissue engineering applications.

### 3.5. Yield and Spectral Characterization of Total Lipid Extracts

The skin and bone of *Caranx hippos* were used as raw materials for lipid extraction. Regarding the extraction of SL and BL by the Soxhlet method (samples referred to as SLs and BLs, respectively), no significant differences (*p* ≥ 0.05) were observed in the yields (14.19 and 17.46% w.b.) between them, indicating that skin and bones are suitable as raw materials when an efficient extraction method is used. The SL sample obtained by extraction using the Folch method (SLf) had a lower yield (4.22% w.b.). The Soxhlet method utilizes petroleum ether, a nonpolar solvent that effectively extracts nonpolar lipids, such as triglycerides. On the other hand, the Folch method utilizes a polar solvent (methanol) and a nonpolar solvent (chloroform), which enables the extraction of lipids, including triglycerides, phospholipids, and glycolipids. However, the higher yield of the Soxhlet method can be attributed to the longer extraction time and continuous application of heat during the process, which promotes the solubilization and extraction of lipids present in fish skin.

Comparing the FTIR spectra of SLs, BLs, and SLf, it was observed that SLf better preserved the *cis* double bonds at 3013, 1657, and 926 cm^−1^ (Figure 9), while the Soxhlet method could induce lipid oxidation and degradation of heatliable compounds, such as PUFA, probably due to more prolonged exposure to the boiling temperature [66]. Therefore, the spectra showed some differences, especially in the fingerprint region (1240–650 cm^−1^).

### 3.6. Total Lipid Fatty Acids

In the SLs, BLs, and SLf samples, SFAs were predominant in all samples (Table 8), followed by MUFAs and PUFAs, which is consistent with the results reported by Hernández-Martínez et al. (2016) [67] in their analysis of the fatty acid profile in *Caranx hippos* fillet, where the SFA:MUFA: PUFA ratio was on average 1:0.67:0.61; however, there are differences between the proportion of fatty acids in the fillet and the *Caranx hippos* waste, especially in PUFA.

In general, the SFA:MUFA:PUFA ratio is a key indicator of the nutritional quality of total lipid content, with a higher proportion of MUFA and PUFA compared to SFA being desirable due to their cardiovascular and metabolic benefits [68].

The FAME of SLs and BLs were compared with the commercial CFO sample and with the SLf sample extracted using the Folch method, which is a cold method. A higher SFA content was observed in SLs compared to SLf. Regarding PUFA, the highest content (*p* < 0.05) was found in SLf. The lower proportion of PUFA in oils obtained using the Soxhlet technique can be attributed to the thermal degradation of these compounds, as they are highly susceptible to oxidation and breakdown under prolonged thermal conditions [66,69]. 

On the other hand, the nonpolar nature of the petroleum ether used in Soxhlet extraction favors the extraction of nonpolar lipids, such as triglycerides. However, it limits the recovery of polar lipids, which may also influence the fatty acid profile compared to the Folch method, which uses a mixture of nonpolar and polar solvents (chloroform/methanol) to allow the release of all classes of lipids.

The SLs and BLs samples have a lower PUFA content than the CFO sold as an omega-3 supplement. In this sense, the results reflect that the supplements are formulated as a source of omega-3, which is why their EPA and DHA content is maximized.

Considerable variability in EPA and DHA content has been reported among different fish species. For example, Mgbechidinma et al. (2023) [15] evaluated the waste of *Scomberomorus sinensis* (EPA: 8.88%, DHA: 18.21%) and *Carassius auratus* (EPA: 6.02%, DHA: 20.42%). López-Puebla et al. (2025) [68] analyzed bones of *Engraulis ringens* (EPA: 14.73%, DHA: 8.24%), *Trachurus murphyi* (EPA: 10.75%, DHA: 13.10%), and *Schizosaccharomyces japonicus* (EPA: 9.47%, DHA: 11.88%). Balikci (2024) [70] documented the fatty acid profiles of *Capoeta sieboldi* (EPA: 12.86%, DHA: 8.26%) and *Capoeta tinca* (EPA: 11.79%, DHA: 8.30%) fillets, emphasizing that variations in composition occur throughout the year. The results show that there is no single species that is ideal for oil extraction.

The SLs, BLs, and SLf samples had a favorable PUFA proportion and ω-3/ω-6 ratio (Table 8), mainly when the process did not involve long extraction times or high temperatures. The results suggest that oils extracted from *Caranx hippos* show potential as functional ingredients, as they can serve as a source of omega-3 fatty acids and can be considered an ingredient for supplement enrichment.

### 3.7. Antioxidant Capacity of Total Lipid Extracts

A significant challenge in using fish oil is its high susceptibility to oxidation, which reduces its stability during storage compared to other types of oils [71]. The evaluation of oils with antioxidant properties enables the assessment of their oxidative stability and protective effect against oxidative processes [35].

Regarding the β-carotene bleaching test, Table 9 indicates that SLf exhibits a higher DR value than CFO. A lower DR value indicates greater antioxidant capacity, as it reflects less loss of β-carotene over time [35]. A higher % inhibition was also obtained with CFO. This result indicates that the commercial sample is a more effective antioxidant against β-carotene degradation. However, SLf shows DR and % inhibition values with no significant difference (*p* ≥ 0.05) compared to an extra-virgin olive oil that was also analyzed (DR = 0.0160 ± 0.0008, % inhibition = 71.01 ± 1.54). These results suggest that SLf has an acceptable protective action against free radicals, delaying the oxidation process. 

It is important to note that SLf is a crude extract, neither purified nor concentrated. This condition influences the effective concentration of antioxidants and their bioavailability; the presence of neutral lipids and other impurities can dilute the observed antioxidant effect.

To date, no research has been reported that evaluates the antioxidant capacity of fish oils or their residues using the β-carotene scavenging assay, so the results obtained may serve as a reference for future studies.

The results obtained in the ABTS and DPPH assays showed comparable antioxidant activity between SLf and CFO, both of which exhibited an ABTS radical inhibition percentage of approximately 33%, with no significant differences between them (*p* ≥ 0.05). In the DPPH assay, CFO showed a slightly higher inhibition percentage compared to SLf. Table 9 also includes the concentrations of the antioxidant trolox required to produce an effect equivalent to that of the samples evaluated by the ABTS or DPPH methods. Both samples had similar trolox equivalent values in the two assays, indicating that they have comparable antioxidant potential. 

Regarding inhibition percentages (Table 9), the β-carotene bleaching assay yielded the highest values, indicating that it measures significantly higher antioxidant capacity (*p* < 0.05) compared to the ABTS and DPPH methods. The latter two methods, although widely used in plant products, may underestimate the antioxidant activity of oils due to their lower affinity for highly lipophilic systems. In the present study, they were reported as a reference.

The inhibition percentages obtained indicate that SLf exhibits a moderate antioxidant capacity. According to some authors, this antioxidant capacity is provided by compounds such as α-tocopherol, γ-tocopherol, vitamin A, and vitamin D. It is also secondary to omega-3 PUFAs, including EPA and DHA fatty acids [36,72].

## 4. Materials and Methods

### 4.1. Raw Material

The crevalle jack (*Caranx hippos*) from the coast of Veracruz (19°3′6.8″ N, 95°59′55.0″ W), in the Gulf of Mexico, was purchased fresh and whole at the "La Nueva Viga" Fish and Seafood Market in Mexico City. The fish was gutted and filleted at the point of sale, and the waste was transferred to the laboratory in a cooler, where the raw material was cleaned by manually removing meat residues. The heads were dismembered to separate the skin and bones from the skull, and the waste was washed with tap water. The skin was cut into pieces measuring approximately 1 cm × 1 cm with scissors. The bones were cut to reduce their size and vacuum-dried at 30 °C for 48 hours. The waste was stored separately in vacuum-sealed polyethylene bags and frozen at −20 °C until analysis. 

### 4.2. Chemical Reagents

Acetic acid (Fermont, Monterrey, Mexico), ethanol, methanol, n-butanol, NaOH, EDTA, NaCl, KOH, HNO_3_, and K_2_S_2_O_8_ (Meyer, Ciudad de Mexico, Mexico) were ACS reagents. Chloroform, methanol, and methylene chloride (JT Baker, Phillipsburg, NJ, USA) were HPLC grade. The FAME standard mixture was from Restek (Bellefonte, PA, USA). Hydroxyproline standard (Merck KGaA, Darmstadt, Germany). β-carotene, DPPH, and ABTS were from Sigma-Aldrich (St. Louis, MO, USA). Nitrogen 5.0, hydrogen 5.0, and extra dry air were from Linde (Guanajuato, Mexico).

### 4.3. Chemical Composition

The chemical composition of *Caranx hippos* by-products was obtained in triplicate. The determination of protein (AOAC method 928.08), lipids (AOAC method 960.39), moisture (AOAC method 950.46), and ash (AOAC method 967.05) contents was performed using standard methods of the AOAC (2002) [73]. The carbohydrate content was calculated by difference. The hydroxyproline content in skin (0.1 g) and bone (1.0 g) of fresh *Caranx hippos*, as well as in freeze-dried collagen (10 mg), was analyzed according to the method reported by Oslan et al. (2022) [12] using a hydroxyproline (trans-4-Hydroxy-L-proline) standard curve between 10 and 60 ppm.

### 4.4. Collagen Extraction

Collagen from the skin and bones of *Caranx hippos* was isolated as described below. The process was carried out at 15°C using solutions at 4°C. Non-collagenous proteins were hydrolyzed using 0.1 N NaOH in a sample: solution ratio of 1:10 (*w*/*v*) in an orbital shaker at 200 rpm for 12 h. The alkaline solution was changed every 6 h. Subsequently, it was rinsed with cold water until a neutral pH was achieved in the wash water. Lipids were removed from the skin using a 10% (*v*/*v*) n-butanol solution in a 1:10 (*w*/*v*) ratio, stirred continuously for 24 h at a speed of 200 rpm. The solution was changed every 12 h. The degreased skin was washed with cold water until the washing water reached a neutral pH. The bone, pretreated with NaOH, was demineralized with 0.5 M sodium ethylenediaminetetraacetic acid (EDTA-Na) (pH 7.4) at a ratio of 1:10 (*w*/*v*) for 48 h, with continuous stirring at a speed of 200 rpm. The solution was changed every 24 h. The decalcified bone was washed several times with cold, running water until the washing water had a neutral pH. Collagen extraction was performed in duplicate using 0.5 M acetic acid solution in a 1:10 (*w*/*v*) ratio, for 24 h with continuous stirring at 200 rpm. The extracts were vacuum filtered with Whatman No. 4 paper. 

The supernatant collagen was precipitated by adding 2.5 M NaCl in the presence of 0.05 M Tris (hydroxymethyl) aminomethane-HCl adjusted to pH 7.5. It was left to stand at 4 °C for 24 h until complete precipitation occurred. The resulting precipitate was recovered by centrifugation at 2500× *g* (4000 rpm) for 30 min. The centrifuged collagen was dissolved in the minimum volume of 0.5 mol/L acetic acid. The solution was dialyzed against 10 volumes of 0.1 M acetic acid using a dialysis membrane with a molecular weight cut-off of 6–8 kDa for 48 h at 4 °C, with solution changes every 12 h. The solution was then dialyzed against 10 volumes of distilled water for 48 h at 4 °C, with water changes every 12 h. The collagen obtained was freeze-dried and stored in a desiccator for further analysis. 

Collagen extraction yield (%) was calculated based on the dry matter of the freeze-dried collagen, compared to the dry weight and wet weight of the fish by-product used for extraction.

### 4.5. Physicochemical Characterization of Collagen

#### 4.5.1. Color Analysis

The color of collagen was measured using a ColorFlex EZ colorimeter (HunterLab, Reston, USA), which was calibrated prior to measurement. Briefly, the values of L* (lightness), a* (+a*, red; −a*, green), and b* (+b*, yellow; −b*, blue) on the surface of the collagen were determined in triplicate, placing 500 mg of freeze-dried collagen in the glass cell. The color of the samples was also characterized according to color index (CI), whiteness (W), and Chroma (C*), defined as:

(1) $$CI=\frac{a^{*}\times 1000}{L^{*}\times b^{*}}$$

(2) $$W=100-\sqrt{{\left(100-L^{*}\right)}^{2}+{\left(a^{*}\right)}^{2}+{\left(b^{*}\right)}^{2}}$$

(3) $$C^{*}={\left(a^{*}\right)}^{2}+{\left(b^{*}\right)}^{2}$$

#### 4.5.2. Zeta Potential

The zeta potential (ζ) was measured with a zeta potential meter ZetaPlus 21471 (Brookhaven Instruments, Holtsville, NY, USA). For analysis, collagen samples were first dissolved in 0.5 M acetic acid at a concentration of 0.062% (*w*/*v*). The pH of the solutions was adjusted from 1 to 10 using 3.0 M potassium hydroxide or 3.0 M nitric acid [31]. The potential values of the samples were obtained in triplicate. The isoelectric point (pI) was determined from a pH value at which the potential is zero. 

#### 4.5.3. Differential Scanning Calorimetry (DSC)—Thermal Analysis

Collagen films from CS and BC were analyzed. The films were prepared from lyophilized collagen solutions in 0.5 M acetic acid at a concentration of 12 mg/mL. The solutions were placed in a magnetic stirrer for 72 h. When the collagen was completely dissolved, 1 mL of the solution was poured into a flexible silicone mold to facilitate demolding. The mold was placed in a desiccator for 48 h to allow the films to dry.

Three milligrams of film were analyzed in a differential scanning calorimeter (DSC) (TA Instruments Waters, model Q2000, Newcastle, DE, USA) in the temperature range of 10 to 130 °C at a heating rate of 5 °C/min for 25 min. A sealed aluminum pan with a capacity of 40 µL, brand TZeroTM, was used. The total denaturation enthalpy was estimated by measuring the area under the curve in the DSC thermogram. Thermal transition temperatures and their corresponding enthalpies were calculated using the TA Instruments Universal Analysis 2000 software, based on the thermogram obtained.

### 4.6. Structural Characterization of Collagen

#### 4.6.1. FTIR Spectra Acquisition

The spectra of freeze-dried collagens and total lipid extracts from crevalle jack’s skin and bone were collected using a Fourier transform infrared (FTIR) spectrometer (PerkinElmer, Frontier, Waltham, MA, USA) equipped with an attenuated total reflectance accessory (ATR) of diamond. FTIR spectra were acquired by placing 200 mg of sample onto ATR, using the wavenumber range of 4000–650 cm^−1^ at a resolution of 4 cm^−1^, averaging 64 scans, according to the methodology of Hernández-Martínez et al. (2013) [74].

#### 4.6.2. UV Spectra Acquisition

The ultraviolet (UV) absorption spectra of the samples were measured from 200 to 400 nm using a UV-Vis spectrophotometer (JENWA, model 6305, Stonte, Staffordshire, UK) by dissolving the lyophilized collagen sample (0.5 mg/mL) in 0.5 M acetic acid. The first-derivative UV spectra were obtained with the software Spectragryph v1.2.16 (https://www.effemm2.de/spectragryph/ (accessed on 18 October 2025) by Dr. Friedrich Menges).

#### 4.6.3. X-Ray Diffraction (XRD)

The freeze-dried collagen samples were analyzed using an X-ray diffractometer (Rigaku, MiniFlex 600, Japan), operating in Bragg–Brentano mode with a copper lamp (Cu Kα, λ = 1.5406 Å) at 40 kV and 15 mA. The diffractogram was obtained according to Reátegui-Pinedo et al. (2022) [60] at a scanning rate of 2°/min in the 2θ range from 5 to 60°, with a step increment of 0.02°. The distance between the crystal planes, known as interlaminar distance (dÅ), was calculated using the Bragg equation [60]:

(4) dÅ=λ2sinθ

where λ = X-ray wavelength (1.54 Å) for copper radiation; θ = Bragg diffraction angle. The dÅ value for the first peak (peak A) represents the distance between molecular chains, while the second peak (peak B) indicates the distance between skeletons, in angstrom units.

#### 4.6.4. Electron Microscopy-Energy-Dispersive X-Ray Spectroscopy (SEM-EDX)

An X-ray detector (Bruker, Quantax 200, Karlsruhe, Germany) coupled to an environmental scanning electron microscope (Zeiss EVO LS10, Oberkochen, Germany) was used to perform elemental microanalysis of the collagen samples. For the analysis, the samples were analyzed with an acceleration voltage of 30 kV, a spot size of 720 (dimensionless value), and a count rate of 1000–9000 counts per second (cps).

#### 4.6.5. Structural Characterization by Gel Electrophoresis (SDS-PAGE)

Sodium dodecyl sulfate-polyacrylamide gel electrophoresis (SDS-PAGE) was employed to identify the protein pattern, following the method reported by Wei et al. (2019) [31]. A protein ladder (10–250 kD) (BZ0011G, Bio Basic, NY, USA) was used. Gels were analyzed using GelAnalyzer 23.1.1 (https://www.gelanalyzer.com (accessed on 1 September 2025) by Istvan Lazar Jr., PhD, and Istvan Lazar Sr., PhD, CSc).

### 4.7. Morphological Analysis of Collagen

#### Environmental Scanning Electron Microscopy (ESEM)

The morphological analysis of the lyophilized collagen samples was performed using an environmental scanning electron microscope (ESEM) (JEOL, JSM-5800LV, Nagoya, Japan). Before observation, the samples were placed on aluminum stubs with double-sided copper tape, introduced into the cathodic sputtering equipment, and coated with a thin, uniform layer of gold (<10 nm) on their surface. The samples were observed directly under a microscope with an acceleration voltage of 15 kV. The images were obtained in RGB scale. To evaluate the morphology of the lyophilized collagens, SC and BC, micrographs obtained using ImageJ software (version 1.54p, National Institutes of Health, Bethesda, MD, USA) were analyzed. An analysis of the size of visible pores (diameter µm) and an estimation of surface porosity (%) were performed, parameters reported by López (2019) [65].

### 4.8. Lipid Extraction

Lipids from the skin and bones of crevalle jack (*Caranx hippos*) were extracted using the Soxhlet [73] and Folch (1956) [75] methods to determine their fatty acid profile. The lipids were extracted using the Folch method to quantify their antioxidant activity.

It is essential to note that the Folch and Soxhlet methods were employed exclusively in this study for analytical purposes. To produce edible oil or nutritional supplements, safer and more appropriate techniques are required, such as supercritical fluid extraction or cold pressing, which avoid the use of toxic solvents and are more suitable for human consumption.

### 4.9. Fatty Acid Methyl Ester Determination of Total Lipid Extracts

Aliquots of the whole lipid extract of skin or bone were used to prepare and analysed fatty acid methyl esters (FAMEs) according to Hernández-Martínez et al. (2013) [74] in a PerkinElmer gas chromatograph (Clarus 500, PerkinElmer^®^, Shelton, CT, USA) with flame ionization detector (FID), and an Rtx^®^-2330 column (105 m × 0.25 mm i.d. × 0.20 mm film thickness) (Restek, Bellefonte, PA, USA). Nitrogen was used as the carrier gas at a pressure of 276 kPa (40 psi), and the split ratio was 1:20. The results were recorded as the percentage of the peak area. Fatty acids of samples were identified with standard FAMEs mixtures (Restek 35066 marine and Restek 35077). Each sample was analyzed in triplicate.

### 4.10. Antioxidant Capacity Determination of Total Lipid Extract

For extraction, 50 mg of sample and 5 mL of solvent were mixed in a vortex for 20 min at room temperature. The mixture was then centrifuged at 2500× *g* (4000 rpm) for 10 min, and the supernatant was recovered. The sediment was treated one more time under the same procedure. The antioxidant capacity was quantified by (1) a hydrogen atom transfer-based assay, the β-carotene bleaching assay, using the methodology of Kabouche et al. (2007) [76]; and (2) two electron transfer-based assays, the DPPH and ABTS assays, according to Rufino et al. (2010), using Trolox as a positive control [77]. The AC was calculated as a percentage inhibition. 

For the β-carotene bleaching assay, the β-carotene degradation rate (DR) was also calculated using the equation [69]:

(5) DR=ln(a/b)t

where a is the absorbance of the emulsion at 0 min, b is the absorbance at 45 min of reaction, and t is the time elapsed from the start of the experiment to the measurement point. The negative control was the reaction mixture (β-carotene, linoleic acid, tween 20, water, methanol, dichloromethane) without the lipid extract. 

### 4.11. Statistical Analysis

The mean values obtained were analyzed by one-way analysis of variance (ANOVA) with Tukey’s test (*p* < 0.05). Each test was performed in triplicate. Minitab Statistical Software version 17 (State College, PA, USA) was used for the analyses.

## 5. Conclusions

The extraction of native collagen and total lipid extract from the skin and bones of crevalle jack (*Caranx hippos*) presents a valuable opportunity to reduce waste that is commonly discarded. By transforming these materials into high-value resources, the use of fishery resources can be optimized and the circular economy promoted.

One advantage of collagen from *Caranx hippos* is the low price of the fish ($2.20 to $5.30 USD/kg) and the wide availability of the raw material. The collagen yield is comparable to other sources but could be optimized.

The SC and BC obtained are collagen type I; they retain the integrity of the triple helix structure and have good thermal stability above 37 °C. SC has greater crystallinity, and BC has greater porosity. The pI is low (4.9) compared to that reported for other sources, which can be as high as 7.2, making SC and BC collagen stable at physiological pH.

The physicochemical, structural, and morphological characterization of native collagen reveals that this biomaterial possesses properties suitable for potential applications in the biomedical industry, for example, the creation of scaffolds. In cosmetics, film-forming properties and the hydrophilic nature of collagen, previously reported, contribute to the development of creams, gels, and masks with high moisturizing action. Other activities are anti-wrinkling, UV radiation protectors, and maintaining a healthy skin microbiome and skin barrier. These applications can be explored in further studies. 

On the other hand, *Caranx hippos* is also a source of valuable lipids. The total lipid extracts contain from 6.97 to 13.82% omega-3 fatty acids, relative to the total fatty acids; in addition to possessing antioxidant capacity, they make them a promising alternative for applications in the food or nutraceutical industry. However, fish lipids can also be used in cosmetics, as part of liposomes. The practical significance of obtaining collagen and lipids from *Caranx hippos* is focused on these fields.

## Figures and Tables

**Table 1:** Chemical and physical properties of collagen from the skin and bone of crevalle jack (*Caranx hippos*).

	SC	BC
Moisture (g/100 g)	1.30 ± 0.51 ^a^	0.91 ± 0.42 ^a^
Ash (g/100 g)	0.03 ± 0.01 ^b^	0.21 ± 0.37 ^a^
Hydroxyproline (mg Hyp/g collagen)	19.02 ± 1.31 ^a^	15.47 ± 1.65 ^b^
L*	62.66 ± 0.01 ^b^	86.48 ± 0.01 ^a^
a*	1.7 ± 0.01 ^a^	−3.26 ± 0.03 ^b^
b*	15.16 ± 0.01 ^b^	20.95 ± 0.06 ^a^
CI	1.79 ± 0.01 ^a^	−1.80 ± 0.02 ^b^
W	59.67 ± 0.02 ^b^	78.86 ± 0.05 ^a^
C*ab	15.25 ± 0.01 ^b^	21.20 ± 0.03 ^a^

Results are expressed as mean ± SD (*n* = 3). Means in the same row with different superscripts indicate significant differences (*p* < 0.05). SC, collagen from crevalle jack skin; BC, collagen from crevalle jack bone; L*, lightness, a*, red-green axis; b*, yellow-blue axis; CI, color index; W, whiteness; C*, chroma.

**Table 2:** Elemental composition of collagen from the skin and bone of crevalle jack (*Caranx hippos*).

Element wt.%	SC	BC
Carbon (C)	58.43 ± 5.31	64.42 ± 3.91
Oxygen (O)	22.99 ± 3.82	21.99 ± 3.26
Nitrogen (N)	15.0 ± 2.38	12.25 ± 0.76
Tantalum (Ta)	1.27 ± 1.18	0.43 ± 0.49
Chlorine (Cl)	0.60 ± 0.47	0.18 ± 0.14
Iron (Fe)	0.32 ± 0.00	0
Copper (Cu)	0.29 ± 0.30	0.058 ± 0.10
Sulfur (S)	0.25 ± 0.21	0.18 ± 0.15
Fluorine (F)	0.23 ± 0.15	0.29 ± 0.00
Rhodium (Rh)	0.22 ± 0.09	0.099 ± 0.17
Lead (Pb)	0.18 ± 0.16	0
Silicon (Si)	0.096 ± 0.19	0
Potassium (K)	0.059 ± 0.05	0.048 ± 0.08
Sodium (Na)	0.049 ± 0.03	0.039 ± 0.06

Results are expressed as mean ± SD (*n* = 3). wt.%, percentage by weight; SC, skin collagen; BC, bone collagen.

**Table 3:** Results of differential scanning calorimetry performed on collagen films from the skin and bone of crevalle jack (*Caranx hippos*).

Films	Thermal Transition 1 (Td), °C	ΔH Enthalpy of Td, J/g	Thermal Transition 2 (T2), °C	ΔH Enthalpy of T2, J/g
SC	↓ 53.40 ± 1.28 ^a^	1.005 ± 0.153	↓ 82.39± 0.65 ^b^	2.19 ± 0.28
BC	↓ 46.88 ± 0.98 ^b^	1.428 ± 0.367	↑ 108.48 ± 0.01 ^a^	33.17 ± 3.487

Results are expressed as mean ± SD (*n* = 3). Means with different lower-case letters in the same column indicate significant differences (*p* < 0.05). SC, skin collagen; BC, bone collagen. n.d., not detected; ↓ Endothermic process; ↑ Exothermic process.

**Table 4:** Positions of bands in FTIR spectra and their assignments for collagen from the skin and bone of crevalle jack (*Caranx hippos*).

Region	SC	BC	Assignments	References
	Wavenumber (cm^−1^)			
Amide A	3299 ± 4.31 ^a^	3307 ± 0.35 ^a^	N-H asymmetric stretching	[29]
-	3077 ± 1.24 ^a^	3077 ± 0.70 ^a^	N–H stretching overtone of amide II	[29]
-	3013 ± 0.01 ^a^	3012 ± 0.02 ^a^	C=CH stretching	[29]
-	2954 ± 0.11 ^a^	2955 ± 0.20 ^a^	-CH_3_ asymmetric stretching	[29]
Amide B	2924 ± 5.72 ^a^	2922 ± 3.28 ^a^	-CH_2_ asymmetric stretching	[12,27]
-	2872 ± 0.03 ^a^	2872 ± 0.02 ^a^	-CH_3_ symmetric stretching	[29]
-	2856 ± 0.00 ^a^	2856 ± 0.00 ^a^	-CH_2_ symmetric stretching	[29]
-	1744 ± 0.15 ^a^	1743 ± 0.12 ^a^	C=O stretching	[29]
Amide I	1635 ± 0.60 ^a^	1636 ± 2.82 ^a^	C=O stretching in peptide bond/Hydrogen bond coupled with COO-	[12,28,30]
Amide II	1544 ± 0.23 ^a^	1543 ± 0.90 ^a^	N-H bending/C-N stretching	[27,29]
-	1454 ± 2.82 ^a^	1455 ± 0.21 ^a^	N-H bending/-CH_3_ asymmetric bending	[29]
-	1393 ± 2.05 ^a^	1392 ± 0.56 ^a^	-CH_3_ symmetric bending/COO- symmetrical stretching	[29,31]
-	1337 ± 0.46 ^a^	1336 ± 0.35 ^a^	COO-symmetric stretching/-CH_2_ wagging	[12,30]
Amide III	1238 ± 0.88 ^a^	1237 ± 0.64 ^a^	C-N stretching/N-H in-plane bending/-CH_2_ wagging/CO stretching	[29,32]
-	1203 ± 0.05 ^a^	1199 ± 0.03 ^a^	-	-
-	1160 ± 0.83 ^a^	1160 ± 0.40 ^a^	C-N stretching	[29]
-	1115 ± 0.39 ^a^	1114 ± 0.05 ^a^	-	-
-	1082 ± 0.15 ^a^	1085 ± 0.35 ^a^	C-O stretching	[30]
-	972 ± 0.23 ^a^	973 ± 0.42 ^a^	C-C stretching	[29]
-	922 ± 0.2 ^a^	921 ± 0.05 ^a^	C-C stretching	[29]
-	849 ± 0.04 ^a^	850 ± 0.01 ^a^	C-N stretching	[29]

Results are expressed as mean ± SD (*n* = 3). Means with different lower-case letters in the same row indicate significant differences (*p* < 0.05). SC, skin collagen; BC, bone collagen.

**Table 5:** Molecular weights of the bands of collagen from the skin and bones of crevalle jack (*Caranx hippos*).

Freeze-Dried Samples	Band 1, kDa β dimer	Band 2, kDa Chain α1	Band 3, kDa Chain α2
SC	260	141	106
BC	243	139	114

SC, skin collagen; BC, bone collagen; kDa, kilodaltons.

**Table 6:** Porosity analysis of collagen from the skin and bones of crevalle jack (*Caranx hippos*).

Freeze-Dried Samples	Total Number of Pores (Per Micrograph)	Pore Diameter (µm)	Area Occupied by Pores (%)
SC	1539 ± 329 ^a^	32.87 ± 12.13 ^a^	41.28 ± 23.43 ^a^
BC	1814 ± 696 ^a^	29.94 ± 10.66 ^a^	38.84 ± 15.23 ^a^

The values correspond to the average of representative SEM micrographs per sample ± SD (*n* = 3). Means with different lower-case letters in the same column are significantly different (*p* < 0.05). The number of pores represents the total detected by micrography. Total area analyzed by micrography: 3,008,273 µm^2^ (≈ 3.008 mm^2^). SC, skin collagen; BC, bone collagen.

**Table 7:** Positions of bands in FTIR spectra and their assignments for total lipid extracts from the skin and bone of crevalle jack (*Caranx hippos*).

SLf	SLs	BLs	Assignments	Reference
**Wavenumber ( cm^−1^)**				
3385 ± 0.46 ^b^	3475 ± 0.89 ^a^	3475 ± 0.11 ^a^	O-H stretching of water traces	[29]
3013 ± 0.02 ^a^	3012 ± 0.02 ^a^	3011 ± 0.04 ^a^	=C-H stretching	[29]
2955 ± 0.51 ^a^	2954 ± 0.01 ^a^	2954 ± 0.03 ^a^	-CH_3_ asymmetric stretching	[29]
2922 ± 0.19 ^a^	2923 ± 0.01 ^a^	2922 ± 0.06 ^a^	-CH_2_ asymmetric stretching	[29]
2852 ± 0.15 ^a^	2853 ± 0.01 ^a^	2853 ± 0.04 ^a^	-CH_2_ symmetric stretching	[29]
1742 ± 0.11 ^a^	1743 ± 0.02 ^a^	1742 ± 0.01 ^a^	C=O stretching in triglycerides, phospholipids	[29]
1657 ± 0.54 ^a^	1659 ± 0.39 ^a^	1659 ± 0.59 ^a^	C=C stretching in *cis* fatty acids	[29]
1553 ± 0.67	-	-	-	-
1465 ± 0.03 ^a^	1464 ± 0.06 ^a^	1464 ± 0.01 ^a^	-CH_2_ and -CH_3_ bending	[15,29]
1416 ± 0.50 ^a^	1417 ± 0.04 ^a^	1417 ± 0.11 ^a^	COO- symmetric stretching in carboxylate group/-CH_2_ bending	[29]
1378 ± 0.02 ^a^	1377 ± 0.01 ^a^	1377 ± 0.01 ^a^	-CH_3_ symmetric bending	[29]
1219 ± 0.54 ^c^	1235 ± 0.36 ^b^	1237 ± 0.06 ^a^	C-O stretching in trigliceridos/-CH_2_ bending	[15]
1173 ± 0.38	-	-	C-O stretching	[15]
-	1156 ± 0.03 ^a^	1159 ± 0.13 ^a^	C-O stretching	[29]
-	1115 ± 0.68 ^a^	1116 ± 0.00 ^a^	C-O stretching	[29]
1093 ± 0.54 ^a^	1097 ± 0.00 ^a^	1097 ± 0.00 ^a^	C-O stretching	[15]
1061 ± 0.23	-	-	-	-
971 ± 0.04 ^a^	971 ± 0.02 ^a^	972 ± 0.02 ^a^	C-N asymmetric stretching	[29]
926 ± 0.14	-	-	=CH_2_ bending out of plane	[15]
890 ± 0.16	-	-	-CH_2_ rocking	[29]
837 ± 0.06	-	-	=CH_2_ wagging	[15]
758 ± 0.43	-	-	-	-
721 ± 0.29 ^a^	721 ± 0.02 ^a^	721 ± 0.01 ^a^	CH_2_ rocking	[15,29]

Results are expressed as mean ± SD (*n* = 3). Means with different lower-case letters in the same row indicate significant differences (*p* < 0.05). SLs, skin lipids extracted by Soxhlet; BLs, bone lipids extracted by Soxhlet; SLf, skin lipids extracted by Folch.

**Table 8:** Fatty acid profile of total lipid extracts from the skin and bones of crevalle jack (*Caranx hippos*) and a commercial oil sample.

Fatty Acid (% of Total Fatty Acids)	SLs	BLs	SLf	CFO
C6:0	n.d.	n.d.	n.d.	0.68 ± 0.01
C8:0	n.d.	n.d.	n.d.	0.10 ± 0.02
C12:0	n.d.	n.d.	n.d.	0.08 ± 0.03
C14:0	5.52 ± 0.39 ^a^	5.60 ± 0.49 ^a^	3.04 ± 0.02 ^b^	6.42 ± 0.96
C16:0	28.64 ± 0.60 ^a^	28.74 ± 0.79 ^a^	26.78 ±1.24 ^a^	15.31 ± 2.04
C17:0	1.84 ± 0.005 ^ab^	1.93 ± 0.18 ^a^	1.66 ± 0.03 ^b^	0.52 ± 0.04
C18:0	11.81 ± 0.28 ^a^	11.42 ± 0.17 ^a^	11.63 ± 0.09 ^a^	3.17 ± 0.05
C20:0	0.99 ± 0.04 ^a^	0.91 ± 0.03 ^a^	0.75 ± 0.01 ^b^	1.3 ± 0.16
C21:0	0.26 ± 0.004 ^b^	0.25 ± 0.02 ^b^	0.38 ± 0.02 ^a^	0.08 ± 0.02
C22:0	0.59 ± 0.03 ^a^	0.50 ± 0.11 ^ab^	0.41 ± 0.01 ^b^	2.81 ± 0.11
C23:0	0.15 ± 0.03 ^a^	0.15 ± 0.03 ^a^	0.17 ± 0.02 ^a^	0.23 ± 0.15
C24:0	0.73 ± 0.02 ^a^	0.67 ± 0.08 ^a^	0.51 ± 0.02 ^b^	3.74 ± 0.85
Σ SFA	50.53 ± 0.60 ^a^	50.19 ± 0.71 ^a^	45.33 ± 1.04 ^b^	34.44 ± 2.64
C14:1 (n-5)	1.44 ± 0.05 ^a^	1.38 ± 0.18 ^a^	0.75 ± 0.01 ^b^	0.45 ± 0.04
C16:1 (n-7)	4.91 ± 0.12 ^ab^	5.21 ± 0.13 ^a^	4.55 ± 0.19 ^b^	8.56 ± 0.76
C17:1 (n-7)	0.68 ± 0.01 ^a^	0.56 ± 0.18 ^a^	0.62 ± 0.01 ^a^	1.13 ± 0.01
C18:1t (n-9)	0.37 ± 0.01 ^a^	0.23 ± 0.22 ^a^	0.38 ± 0.005 ^a^	1.20 ± 0.02
C18:1c9 (n-9)	18.12 ± 0.34 ^a^	19.11 ± 1.62 ^a^	17.61 ± 0.03 ^a^	10.81 ± 1.46
C18:1c14 (n-7)	2.96 ± 0.05 ^a^	2.14 ± 1.30 ^a^	2.18 ± 0.10 ^a^	1.52 ± 0.08
C20:1c	0.64 ± 0.02 ^a^	0.50 ± 0.13 ^a^	0.23 ± 0.01 ^a^	0.22 ± 0.05
C22:1n (n-9)	0.15 ± 0.01 ^a^	0.16 ± 0.005 ^a^	0.13 ± 0.01 ^a^	0.28 ± 0.01
C24:1 (n-9)	0.59 ± 0.05 ^a^	0.60 ± 0.06 ^a^	0.98 ± 0.03 ^a^	0.25 ± 0.01
Σ MUFA	31.24 ± 0.28 ^a^	31.24 ± 0.28 ^a^	27.43 ± 0.08 ^b^	24.42 ± 0.78
C18:2t (n-6)	0.56 ± 0.02 ^a^	0.56 ± 0.10 ^a^	0.38 ± 0.04 ^a^	0.26 ± 0.06
C18:2c (n-6)	1.44 ± 0.01 ^a^	1.53 ± 0.02 ^a^	0.83 ± 0.06 ^b^	2.09 ± 0.51
C18:3c 6,9 (n-6)	0.11 ± 0.02 ^b^	0.17 ± 0.004 ^a^	0.11 ± 0.005 ^b^	0.11 ± 0.01
C18:3c9,12 (n-3)	1.77 ± 0.07 ^a^	1.87 ± 0.28 ^a^	0.88 ± 0.01 ^b^	3.10 ± 0.27
C20:2 (n-6)	0.61 ± 0.03 ^a^	0.63 ± 0.12 ^a^	0.27 ± 0.005 ^b^	0.01 ± 0.01
C20:3c8 (n-6)	0.17 ± 0.01 ^a^	0.09 ± 0.02 ^c^	0.13 ± 0.00 ^b^	0.13 ± 0.01
C20:3c11(n-9)	0.20 ± 0.02 ^a^	0.19 ± 0.07 ^a^	0.13 ± 0.01 ^a^	0.09 ± 0.01
C20:4 (n-6)	1.50 ± 0.05 ^b^	1.57 ± 0.01 ^b^	2.80 ± 0.13 ^a^	3.74 ± 0.85
C20:5 EPA (n-3)	1.26 ± 0.05 ^b^	1.62 ± 0.35 ^b^	3.16 ± 0.04 ^a^	17.59 ± 0.28
C22:2 (n-6)	0.35 ± 0.01 ^a^	0.37 ± 0.05 ^a^	0.36 ± 0.01 ^a^	0.79 ± 0.07
C22:6 DHA (n-3)	3.94 ± 0.20 ^b^	4.57 ± 0.58 ^b^	9.78 ± 0.03 ^a^	11.23 ± 0.76
Σ PUFA	11.93 ± 0.42 ^b^	13.15 ± 1.50 ^b^	18.70 ± 0.36 ^a^	39.14 ± 1.12
n-3/n-6 ratio	1.47:1	1.64:1	2.85:1	4.54:1
SFA:MUFA:PUFA	1:0.62:0.24	1:0.62:0.26	1:0.6:0.4	1:0.71:1.14

Results are expressed as mean ± SD (*n* = 3). Means with different lower-case letters in the same row indicate significant differences (*p* < 0.05). n.d., not detected; SLs, skin lipids extracted by Soxhlet; BLs, bone lipids extracted by Soxhlet; SLf, skin lipids extracted by Folch; CFO, commercial fish oil; MUFA, monounsaturated fatty acid; PUFA, polyunsaturated fatty acid; SFA, saturated fatty acid.

**Table 9:** Antioxidant capacity of total lipid extract from the skin of crevalle jack (*Caranx hippos*) and commercial oil sample.

Assay		Sample	
		SLf	CFO
β-carotene bleaching	% inhibition ^1^	70.83 ± 0.18 ^b^	80.71 ± 0.64 ^a^
	DR	0.0161 ± 0.0001 ^a^	0.0106 ± 0.0003 ^b^
ABTS	% inhibition	32.70 ± 3.61 ^a^	32.90 ± 1.14 ^a^
	Concentration ^2^	0.039 ± 0.001 ^a^	0.039 ± 0.005 ^a^
DPPH	% inhibition	19.60 ± 0.69 ^b^	19.98 ± 0.79 ^a^
	Concentration ^2^	0.0061 ± 0.001 ^b^	0.0087 ± 0.001 ^a^

Results are expressed as mean ± SD (*n* = 3). Means with different lower-case letters in the same row indicate significant differences (*p* < 0.05). SLf, skin lipids extracted by Folch; CFO, commercial fish oil; DR, degradation rate of β-carotene; ^1^ percentage of inhibition of β-carotene bleaching rate, using oil at a concentration of 1 mg/mL and a time of 45 min. ^2^ concentration in µMoles equivalent of trolox per mL.

## Data Availability

All data generated or analyzed during this study are available upon request fthe corresponding author.

## Supplementary Materials

The following supporting information can be downloaded at: https://www.mdpi.com/article/10.3390/md23110432/s1, Figure S1: First-derivative UV spectra of collagens from *Caranx hippos*. SC, skin collagen; BC, bone collagen.

## References

[1] FAO (2024). The State of World Fisheries and Aquaculture 2024. Blue Transformation in Action.

[2] Bachis E. (2024). By-Product.

[3] United Nations (UN) (2025). 17 Goals to Transform Our World.

[4] Rotter A., Barbier M., Bertoni F., Bones A.M., Cancela M.L., Carlsson J., Carvalho M.F., Cegłowska M., Chirivella-Martorell J., Conk-Dalay M. (2021). The essentials of marine biotechnology. Front. Mar. Sci..

[5] Asharaf F., Rajasree S.R. R., Rajan R. (2024). Bioconversion of Eel Skin Waste into Valuable Collagen: Isolation, Spectral Characterization, and Biocompatibility Assessment. Waste Biomass Valorization.

[6] Shekhter A.B., Fayzullin A.L., Vukolova M.N., Rudenko T.G., Osipycheva V.D., Litvitsky P.F. (2019). Medical applications of collagen and collagen-based materials. Curr. Med. Chem..

[7] Meyer M. (2019). Processing of collagen based biomaterials and the resulting materials properties. Biomed. Eng. Online.

[8] Lozano Teruel J.A. (2005). Bioquímica y Biología Molecular Para Ciencias de la Salud.

[9] Lu W.C., Chiu C.S., Chan Y.J., Mulio A.T., Li P.H. (2023). Characterization and biological properties of marine by-product collagen through ultrasound-assisted extraction. Aquac. Rep..

[10] Rajabimashhadi Z., Gallo N., Salvatore L., Lionetto F. (2023). Collagen derived from fish industry waste: Progresses and challenges. Polymers.

[11] Prajaputra V., Isnaini N., Maryam S., Ernawati E., Deliana F., Haridhi H.A., Fadli N., Karina S., Agustina S., Nurfadillah N. (2024). Exploring marine collagen: Sustainable sourcing, extraction methods, and cosmetic applications. S. Afr. J. Chem. Eng..

[12] Oslan S.N.H., Shapawi R., Mokhtar R.A.M., Noordin W.N.M., Huda N. (2022). Characterization of Acid- and Pepsin-Soluble Collagen Extracted from the Skin of Purple-Spotted Bigeye Snapper. Gels.

[13] MarkNtel Advisors (2025). Global Marine Collagen Market Research Report: Forecast (2024–2030).

[14] Alfio V.G., Manzo C., Micillo R. (2021). From fish waste to value: An overview of the sustainable recovery of omega-3 for food supplements. Molecules.

[15] Mgbechidinma C.L., Zheng G., Baguya E.B., Zhou H., Okon S.U., Zhang C. (2023). Fatty acid composition and nutritional analysis of waste crude fish oil obtained by optimized milder extraction methods. Environ. Eng. Res..

[16] Swetha N., Mathanghi S.K. (2024). Towards sustainable omega-3 fatty acids production—A comprehensive review on extraction methods, oxidative stability and bio-availability enhancement. Food Chem. Adv..

[17] Barta D.G., Coman V., Vodnar D.C. (2021). Microalgae as sources of omega-3 polyunsaturated fatty acids: Biotechnological aspects. Algal Res..

[18] Grand View Research (2025). Omega 3 Supplements Market Size Report: Forecast (2025–2030).

[19] Smith-Vaniz W.F., Carpenter K.E. (2007). Review of the crevalle jacks, Caranx hippos complex (Teleostei: Carangidae), with a description of a new species from West Africa. Fish. Bull..

[20] Pacheco C., Cusba J., Bustamante C. (2025). Fishery biology of the Crevalle jack *Caranx hippos* (L) from the Colombian Caribbean. Fish. Res..

[21] PROFECO, Procuraduría Federal del Consumidor (2024, marzo) *De Temporada. Pescados y Mariscos* [Seasonal. Fish and seafood]. Revista del Consumidor. https://www.profeco.gob.mx/revista/RevistaDelConsumidor_565_MARZO_2024.pdf.

[22] Lopes C., Antelo L.T., Franco-Uría A., Alonso A.A., Pérez-Martín R. (2015). Valorisation of fish by-products against waste management treatments—Comparison of environmental impacts. Waste Manag..

[23] Islam J., Mis Solval K.E. (2025). Recent Advancements in Marine Collagen: Exploring New Sources, Processing Approaches, and Nutritional Applications. Mar. Drugs.

[24] Alcolea Ersinger V.F., Lamas D., Massa Á. (2025). A review of marine collagens: Approaches on extractions, applications, market, and future trends. Environ. Sci. Pollut. Res..

[25] Mamat M.N.I.B., Abdul Rahman H., Mohd Razali N.S., Syed Hussain S.S., Kasim K.F., Sofian-Seng N.S. (2025). A Review on Fish Oil Extraction from Fish by-Product as Sustainable Practices and Resource Utilization in the Fish Processing Industry. Sains Malays..

[26] Kalkan E., Keskin Çavdar H., Maskan M. (2025). Health impacts and innovative extraction methods of fish oil: A review. Eur. J. Lipid Sci. Technol..

[27] Martins E., Fernandes R., Alves A.L., Sousa R.O., Reis R.L., Silva T.H. (2022). Skin byproducts of *Reinhardtius hippoglossoides* (Greenland Halibut) as ecosustainable source of marine collagen. Appl. Sci..

[28] Seixas M.J., Martins E., Reis R.L., Silva T.H. (2020). Extraction and characterization of collagen from elasmobranch byproducts for potential biomaterial use. Mar. Drugs.

[29] Socrates G. (2006). Infrared and Raman Characteristic Group Frequencies: Tables and Charts.

[30] Johny L.C., Vijaykumar M., Kudre T.G., Surech P.V. (2022). Malabar sole (*Cynoglossus macrostomus*) skin as promising source of type I acid and pepsin solubilized collagens with potential bioactivity. J. Food Sci. Technol..

[31] Wei P., Zheng H., Shi Z., Li D., Xiang Y. (2019). Isolation and characterization of acid-soluble collagen and pepsin-soluble collagen from the skin of hybrid sturgeon. J. Wuhan Univ. Technol.-Mater. Sci. Ed..

[32] Zhou Y., Liang J., Zhang Y., Zhang H., Chang S.K., Hong H., Luo Y., Tan Y. (2024). Silver carp swim bladder collagen derived from deep eutectic solvents: Enhanced solubility against pH and NaCl stresses. Int. J. Biol. Macromol..

[33] Matarsim N.N., Jaziri A.A., Shapawi R., Mokhtar R.A.M., Noordin W.N.M., Huda N. (2023). Type I collagen from the skin of Barracuda (*Sphyraena* sp.) prepared with different organic acids: Biochemical, microstructural and functional properties. J. Funct. Biomater..

[34] Li J., Wang M., Qiao Y., Tian Y., Liu J., Qin S., Wu W. (2018). Extraction and characterization of type I collagen from skin of tilapia (*Oreochromis niloticus*) and its potential application in biomedical scaffold material for tissue engineering. Process Biochem..

[35] León G.C.D., Reyes Z.P.X. (2017). Estandarización de la Técnica Blanqueamiento del Betacaroteno Para la Evaluación de la Actividad Antioxidante de Extractos Lipofílicos: Plantas Medicinales, Frutos y Microalgas. Bachelor’s Thesis.

[36] Gulcin I. (2020). Antioxidants and antioxidant methods: An updated overview. Arch. Toxicol..

[37] Silva T.H., Moreira-Silva J., Marques A.L., Domingues A., Bayon Y., Reis R.L. (2014). Marine origin collagens and its potential applications. Mar. Drugs.

[38] Martins E., Diogo G.S., Pires R., Reis R.L., Silva T.H. (2022). 3D biocomposites comprising marine collagen and silica-based materials inspired on the composition of marine sponge skeletons envisaging bone tissue regeneration. Mar. Drugs.

[39] Yu D., Chi C.-F., Wang B., Ding G.-F., Li Z.-R. (2014). Characterization of acid-and pepsin-soluble collagens from spines and skulls of skipjack tuna (*Katsuwonus pelamis*). Chin. J. Nat. Med..

[40] Cadar E., Pesterau A.M., Prasacu I., Ionescu A.M., Pascale C., Dragan A.M.L., Sirbu R., Tomescu C.L. (2024). Marine Antioxidants from Marine Collagen and Collagen Peptides with Nutraceuticals Applications: A Review. Antioxidants.

[41] Abbas A.A., Shakir K.A., Walsh M.K. (2022). Functional properties of collagen extracted from catfish (*Silurus triostegus*) waste. Foods.

[42] Emam A.N. (2025). Collagen and collagen-derived materials: Synthesis, structure, classification, fundamental properties and biomedical applications. Discov Appl. Sci..

[43] Salvatore L., Gallo N., Natali M.L., Terzi A., Sannino A., Madaghiele M. (2021). Mimicking the hierarchical organization of natural collagen: Toward the development of ideal scaffolding material for tissue regeneration. Front. Bioeng. Biotechnol..

[44] Jaziri A.A., Shapawi R., Mokhtar R.A.M., Noordin W.N.M., Huda N. (2022). Physicochemical and microstructural analyses of pepsin-soluble collagens derived from lizardfish (*Saurida tumbil* Bloch, 1795) skin, bone and scales. Gels.

[45] Kittiphattanabawon P., Benjakul S., Visessanguan W., Nagai T., Tanaka M. (2005). Characterisation of acid-soluble collagen from skin and bone of bigeye snapper (*Priacanthus tayenus*). Food Chem..

[46] Chinh N.T., Manh V.Q., Trung V.Q., Lam T.D., Huynh M.D., Tung N.Q., Trinh N.D., Hoang T. (2019). Characterization of collagen derived from tropical freshwater carp fish scale wastes and its amino acid sequence. Nat. Prod. Commun..

[47] Morales S.M., Chacón A., Mostue M., Prin J. (2023). Análisis químico de colágeno en piel de cola de atún (*Thunnus atlanticus*) en medio ácido. [Chemical analysis of collagen in tuna tail skin (*Thunnus atlanticus*) in an acid medium]. Revista ESPAMCIENCIA.

[48] Mobarak M.H., Islam M.A., Hossain N., Al Mahmud M.Z., Rayhan M.T., Nishi N.J., Chowdhury M.A. (2023). Recent advances of additive manufacturing in implant fabrication—A review. Appl. Surf. Sci. Adv..

[49] Yue C., Ding C., Xu M., Hu M., Zhang R. (2024). Self-Assembly Behavior of Collagen and Its Composite Materials: Preparation, Characterizations, and Biomedical Engineering and Allied Applications. Gels.

[50] Ahmad M., Benjakul S., Nalinanon S. (2010). Compositional and physicochemical characteristics of acid solubilized collagen extracted from the skin of unicorn leatherjacket (*Aluterus monoceros*). Food Hydrocoll..

[51] Singh P., Benjakul S., Maqsood S., Kishimura H. (2011). Isolation and characterisation of collagen extracted from the skin of striped catfish (*Pangasianodon hypophthalmus*). Food Chem..

[52] Matmaroh K., Benjakul S., Prodpran T., Encarnacion A.B., Kishimura H. (2011). Characteristics of acid soluble collagen and pepsin soluble collagen from scale of spotted golden goatfish (*Parupeneus heptacanthus*). Food Chem..

[53] Kozlowska J., Sionkowska A., Skopinska-Wisniewska J., Piechowicz K. (2015). Northern pike (*Esox lucius*) collagen: Extraction, characterization and potential application. Int. J. Biol. Macromol..

[54] Ge B., Wang H., Li J., Liu H., Yin Y., Zhang N., Qin S. (2020). Comprehensive assessment of Nile tilapia skin (*Oreochromis niloticus*) collagen hydrogels for wound dressings. Mar. Drugs.

[55] Fernandes R.M.T., Couto Neto R.G., Paschoal C.W.A., Rohling J.H., Bezerra C.W.B. (2008). Collagen films from swim bladders: Preparation method and properties. Colloids Surf. B.

[56] Dzorkpata C. (2021). The Effects of NaCl on the Triple Helix Structure of Collagen and the Reinforcement of Tung Oil-Based Polymers with Collagen Peptides. Master’s Thesis.

[57] Vladu A.F., Albu Kaya M.G., Truşcă R.D., Motelica L., Surdu V.A., Oprea O.C., Constantinescu R.R., Cazan B., Ficai D., Andronescu E. (2025). The Role of Crosslinking Agents in the Development of Collagen–Hydroxyapatite Composite Materials for Bone Tissue Engineering. Materials.

[58] Liao W., Guanghua X., Li Y., Shen X.R., Li C. (2018). Comparison of characteristics and fibril-forming ability of skin collagen from barramundi (*Lates calcarifer*) and tilapia (*Oreochromis niloticus*). Int. J. Biol. Macromol..

[59] Uchida D.T., Volnistem A.D.N., Cook M.T., Bruschi M.L. (2025). Effect of the Extraction Methods on the Physicochemical Characteristics of Collagen Derived from Tilapia (*Oreochromis niloticus*) Skin. ACS Omega.

[60] Reátegui-Pinedo N., Salirrosas D., Sánchez-Tuesta L., Quiñones C., Jáuregui-Rosas S.R., Barraza G., Cabrera A., Ayala-Jara C., Martínez R.M., Rolim-Baby A. (2022). Characterization of collagen from three genetic lines (gray, red and F1) of *Oreochromis niloticus* (tilapia) skin in young and old adults. Molecules.

[61] Xiao L., Lv J., Liang Y., Zhang H., Zheng J., Lin F., Wen X. (2023). Structural, physicochemical properties and function of swim bladder collagen in promoting fibroblasts viability and collagen synthesis. LWT.

[62] Tan Y., Chang S.K. (2018). Isolation and characterization of collagen extracted from channel catfish (*Ictalurus punctatus*) skin. Food Chem..

[63] Tapia-Vasquez A.E., Torres-Arreola W., Ezquerra-Brauer J.M., Márquez-Ríos E., Santacruz-Ortega H., Ramírez-Suárez J.C., García-Sánchez G., Suárez-Jiménez G.M. (2025). Spectrometric determination of the collagen crosslinking degree through pyridinoline identification and evaluation of the viscosity properties of *Octopus vulgaris* and *Dosidicus gigas* arm muscles. Appl. Food Res..

[64] Arumugam G.K.S., Sharma D., Balakrishnan R.M., Ettiyappan J.B.P. (2018). Extraction, optimization and characterization of collagen from sole fish skin. Sustain. Chem. Pharm..

[65] López S.E. (2019). Andamios y Biomoléculas de Origen Marino Para Regeneración Tisular del Sistema Osteoarticular. Doctoral Thesis.

[66] Saini R.K., Prasad P., Shang X., Keum Y.S. (2021). Advances in lipid extraction methods—A review. Int. J. Mol. Sci..

[67] Hernández-Martínez M., Gallardo-Velázquez T., Osorio-Revilla G., Castañeda-Pérez E., Uribe-Hernández K. (2016). Characterization of Mexican fishes according to fatty acid profile and fat nutritional indices. Int. J. Food Prop..

[68] López-Puebla S., Arias-Santé M.F., Romero J., Costa de Camargo A., Rincón-Cervera M.Á. (2025). Analysis of Fatty Acid Profile, α-Tocopherol, Squalene and Cholesterol Content in Edible Parts and By-Products of South Pacific Wild Fishes. Mar. Drugs.

[69] Rincón-Cervera M.Á., Villarreall-Rubio M.B., Valenzuela R., Valenzuela A. (2017). Comparison of fatty acid profiles of dried and raw by-products from cultured and wild fishes. Eur. J. Lipid Technol..

[70] Balikci E. (2024). Influence of seasons and fish body parts on fatty acid profile and effect of seasons on proximate composition of Anatolian khramulya (*Capoeta tinca*) and Colchic khramulya (*Capoeta sieboldii*) captured from the Çekerek Dam in Yozgat, Turkey. J. Food Compos. Anal..

[71] Karabayır E.S., Öğütcü M. (2024). Assessment and comparative analysis of the antioxidant capacity of some food waste for fish oils. Grasas Aceites.

[72] Molla M.T.H., Hasan S., Al Bashera M., Kabir B. (2024). Fatty Acid Composition, Antioxidant Activity and Thrombolytic Activity Analysis of Extracted Lipid from *Colisa fasciatus*. J. Sci. Eng. Pap..

[73] Association of Official Analytical Chemists (AOAC) (2005). Official Methods of Analysis.

[74] Hernández-Martínez M., Gallardo-Velázquez T., Osorio-Revilla G., Almaraz-Abarca N., Ponce-Mendoza A., Vásquez-Murrieta M.S. (2013). Prediction of total fat, fatty acid composition and nutritional parameters in fish fillets using MID-FTIR spectroscopy and chemometrics. LWT.

[75] Folch J., Less M., Sloane G. (1956). A simple method for the isolation and purification of total lipides from animal tissues. JCB.

[76] Kabouche A., Kabouche Z., Öztürk M., Kolak U., Topçu G. (2007). Antioxidant abietane diterpenoids from *Salvia barrelieri*. Food Chem..

[77] Rufino M.S.M., Alves R.E., de Brito E.S., Pérez-Jiménez J., Saura-Calixto F., Mancini-Filho J. (2010). Bioactive compounds and antioxidant capacities of 18 non-traditional tropical fruits from Brazil. Food Chem..

