# Supplementary figures and images for: Native Collagen and Total Lipid Extract Obtained from Caranx hyppos By-Products: Characterization for Potential Use in the Biomedical and Nutraceutical Fields
Source: Mar Drugs. 2025 Nov 9;23(11):432. doi: 10.3390/md23110432 (PMC12654203; doi:10.3390/md23110432)

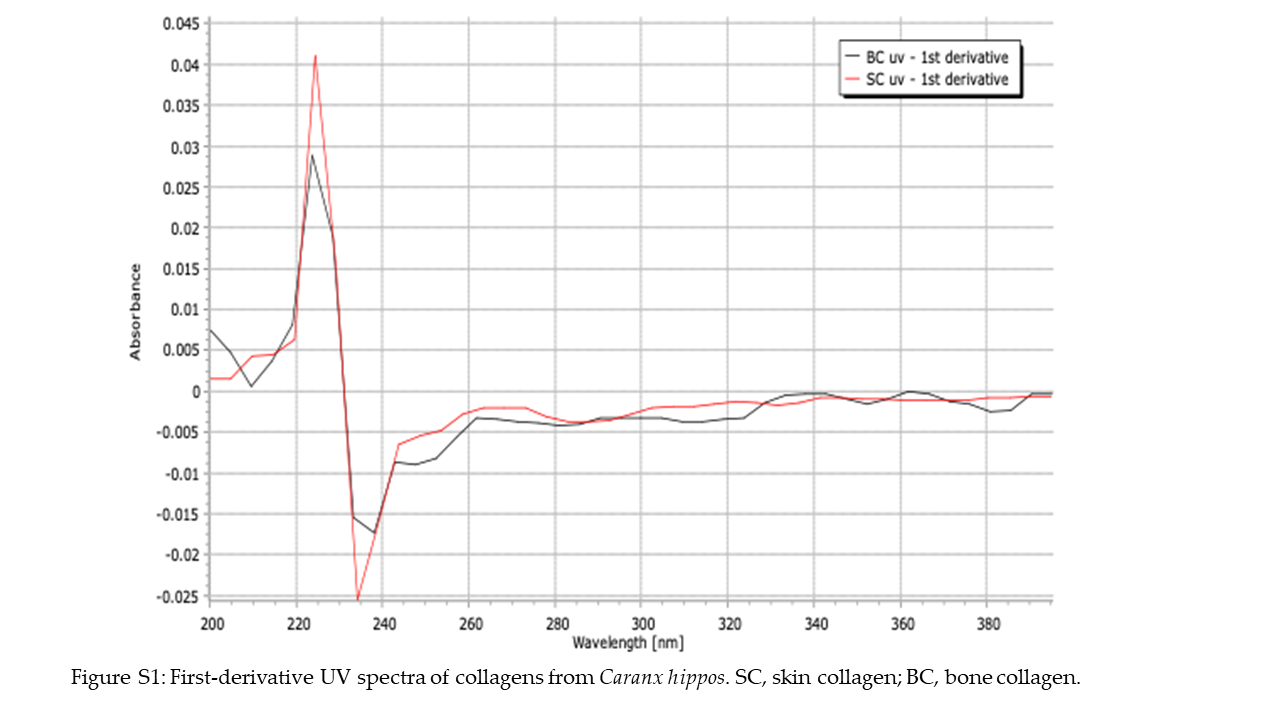

Supplement: Supplementary file 1 [file marinedrugs-23-00432-s001.zip › marinedrugs-3920883-supplementary.png]
